# The effect of preoperative behaviour change interventions on pre- and post-surgery health behaviours, health outcomes, and health inequalities in adults: A systematic review and meta-analyses

**DOI:** 10.1371/journal.pone.0286757

**Published:** 2023-07-05

**Authors:** Mackenzie Fong, Eileen Kaner, Maisie Rowland, Henrietta E. Graham, Louise McEvoy, Kate Hallsworth, Gabriel Cucato, Carla Gibney, Martina Nedkova, James Prentis, Claire D. Madigan

**Affiliations:** 1 NIHR Applied Research Collaboration, North East and North Cumbria, United Kingdom; 2 Population Health Sciences Institute, Newcastle University, Newcastle Upon Tyne, United Kingdom; 3 Centre for Lifestyle Medicine and Behaviour (CLiMB), The School of Sport, Exercise and Health Sciences, Loughborough University, Loughborough, United Kingdom; 4 NIHR Newcastle BRC, Newcastle upon Hospitals NHS Foundation Trust, Newcastle Upon Tyne, United Kingdom; 5 Faculty of Health and Life Sciences, Northumbria University, Newcastle Upon Tyne, United Kingdom; 6 Department of Perioperative and Critical Care Medicine, Freeman Hospital, Newcastle Upon Hospitals NHS Foundation Trust, Newcastle Upon Tyne, United Kingdom; Edith Cowan University - Joondalup Campus: Edith Cowan University, AUSTRALIA

## Abstract

**Background:**

Prehabilitation interventions are being delivered across surgical specialities to improve health risk behaviours leading to better surgical outcomes and potentially reduce length of hospital stay. Most previous research has focused on specific surgery specialities and has not considered the impact of interventions on health inequalities, nor whether prehabilitation improves health behaviour risk profiles beyond surgery. The aim of this review was to examine behavioural Prehabilitation interventions across surgeries to inform policy makers and commissioners of the best available evidence.

**Methods and findings:**

A systematic review and meta-analysis of randomised controlled trials (RCTs) was conducted to determine the effect of behavioural prehabilitation interventions targeting at least one of: smoking behaviour, alcohol use, physical activity, dietary intake (including weight loss interventions) on pre- and post-surgery health behaviours, health outcomes, and health inequalities. The comparator was usual care or no treatment. MEDLINE, PubMed, PsychINFO, CINAHL, Web of Science, Google Scholar, Clinical trials and Embase databases were searched from inception to May 2021, and the MEDLINE search was updated twice, most recently in March 2023. Two reviewers independently identified eligible studies, extracted data, and assessed risk of bias using the Cochrane risk of bias tool. Outcomes were length of stay, six-minute walk test, behaviours (smoking, diet, physical activity, weight change, and alcohol), and quality of life.

Sixty-seven trials were included; 49 interventions targeted a single behaviour and 18 targeted multiple behaviours. No trials examined effects by equality measures. Length of stay in the intervention group was 1.5 days shorter than the comparator (n = 9 trials, 95% CI -2.6 to -0.4, p = 0.01, *I*^*2*^ 83%), although in sensitivity analysis prehabilitation had the most impact in lung cancer patients (-3.5 days). Pre-surgery, there was a mean difference of 31.8 m in the six-minute walk test favouring the prehabilitation group (n = 19 trials, 95% CI 21.2 to 42.4m, *I*^*2*^ 55%, P <0.001) and this was sustained to 4-weeks post-surgery (n = 9 trials, mean difference = 34.4m (95%CI 12.8 to 56.0, *I*^*2*^ 72%, P = 0.002)). Smoking cessation was greater in the prehabilitation group before surgery (RR 2.9, 95% CI 1.7 to 4.8, *I*^*2*^ 84%), and this was sustained at 12 months post-surgery (RR 1.74 (95% CI 1.20 to 2.55, I^2^ 43%, Tau^2^ 0.09, p = 0.004)There was no difference in pre-surgery quality of life (n = 12 trials) or BMI (n = 4 trials).

**Conclusions:**

Behavioural prehabilitation interventions reduced length of stay by 1.5 days, although in sensitivity analysis the difference was only found for Prehabilitation interventions for lung cancer. Prehabilitation can improve functional capacity and smoking outcomes just before surgery. That improvements in smoking outcomes were sustained at 12-months post-surgery suggests that the surgical encounter holds promise as a teachable moment for longer-term behavioural change. Given the paucity of data on the effects on other behavioural risk factors, more research grounded in behavioural science and with longer-term follow-up is needed to further investigate this potential.

## Introduction

Each year approximately 310 million major operations are performed worldwide [1]. Major surgery imposes significant metabolic stress on patients [2], and complication rates following major surgery remain around 20% [3]. A recent paradigm shift has steered focus towards a proactive model of optimising patients’ health and function in the weeks to months leading up to surgery (i.e., the pre-operative period) to improve resilience to surgical stressors and facilitate recovery—a practice that has come to be known as ‘prehabilitation’. As well as better perioperative outcomes for individual patients, prehabilitation may also improve surgery throughput and resource efficiency by promoting earlier discharge from hospital. This is a priority for governments internationally as they continue to deal with surgical waiting lists that have been exacerbated by the Covid-19 pandemic [4,5].

While prehabilitation can involve medical optimisation (e.g., correction of anaemia, medication adjustment [6]) it may also involve behaviour change, leveraging its capacity as a ‘teachable moment’ where patients may be more motivated to adopt risk-reducing health behaviours [7]. Most behavioural programmes under study in previous literature invariably include an exercise component to improve cardiorespiratory fitness and muscular conditioning. There is also evidence that other behavioural health risk factors including poor diet [8,9] (and resulting excess weight [10]), smoking [11–13], and alcohol use [14–16] may heighten the risk of poorer perioperative outcomes, although prehabilitation programmes addressing these behaviours are relatively understudied.

Behavioural prehabilitation offers the possibility of sustained health behaviour change and, subsequently, public health gain [17] given 1) the large number of operations performed, 2) the high prevalence of behavioural health risk factors in surgical populations [18], and 3) that smoking, risky alcohol use, inadequate physical activity, poor diet (and resulting excess weight) are leading causes of preventable ill-health globally [19,20], including conditions that are commonly managed with surgery, e.g. cancers [21]. Longer elective surgery waiting lists in the wake of the COVID-19 pandemic [22] have prompted the Royal Colleges of Anaesthetists and Surgeons and the Centre for Perioperative Care to call for surgery waiting lists to be turned into ‘preparation lists’ [23], with behavioural modification being recognised as an important component [23]. Behavioural modification interventions in surgical populations also have the potential to reduce health inequalities because people from lower socioeconomic backgrounds are disproportionately represented in hospital and surgery populations, and tend to have more prevalent and multiple health risk behaviours [24,25] and related non-communicable disease [24,25]. Therefore, providing behavioural prehabilitation to patients on waiting lists may be more effective at reaching those from lower socioeconomic groups compared to more universal interventions.

Reviews (and a recent umbrella review [26]) of prehabilitation interventions addressing health risk behaviours have been conducted. However, these have focused on just one or two health behaviours [27–29], have been restricted to digital interventions [30] or have focused on special clinical groups (i.e., bariatric surgery [30], patients with alcohol dependency [26]). Further, no previous reviews have sought to examine the impact of prehabilitation interventions by health inequalities. The primary aim of this review was to examine the effect of behavioural Prehabilitation interventions that target physical activity, diet (including weight loss), alcohol use and smoking on pre- and post- surgery health outcomes and health behaviours across surgery specialities. The secondary aim was to examine these outcomes across the socioeconomic spectrum.

## Methods

The systematic review was registered on Prospero (CRD42021249265). This review is reported according to the Preferred Reporting Items for Systematic Reviews and Meta-Analyses (PRISMA) (see S1 File) [31,32]. Protocol amendments and rationale are presented in S1 Table in S1 File.

### Study inclusion criteria

RCTs with adult participants (≥18 years) that evaluated a prehabilitation intervention initiated before surgery that targeted dietary intake, weight loss, physical activity, alcohol use and/or smoking behaviours were eligible for inclusion. We included physical activity interventions that targeted any subtype of physical activity e.g. (both supervised and non-supervised) exercise [33] as in theory, all subtypes have the same aim of increasing cardio-respiratory fitness to improve the resilience for surgery, and can also promote regular, physical activity over the longer-term. There were no limitations on setting (e.g., hospital-based, home-based) or mode of delivery (e.g., digital or in-person). Trials were included if the comparator group received usual care or an intervention that did not focus on behaviour change. Table 1 details the full inclusion and exclusion criteria.

**Table 1:** Review inclusion and exclusion criteria.

	Inclusion	Exclusion
Population	Adults (aged 18 years or over) who have been identified as requiring surgery/surgical procedure and referred to hospital care; those experiencing pre-assessment and awaiting treatment	Alcohol dependent patients; patients awaiting bariatric (weight loss) surgery; patients with major psychiatric conditions/lacking capacity
Intervention	Interventions initiated before surgery and delivered in any setting e.g., primary care, community, hospital; interventions where aim is to improve one or more health behaviour/s i.e., dietary intake (including weight loss), alcohol/tobacco use, physical activity/sedentary behaviour, regardless if explicitly acknowledged as an intervention target; any mode of delivery e.g., digital or in-person.	Exercise interventions that aim to strengthen a specific set of muscles rather than promote whole-body/general resistance training e.g., inspiratory muscle training; interventions where the diet component only involves dietary supplementation e.g., protein drinks
Comparator	Usual care, no intervention, or an intervention that does not aim to modify health behaviours (diet, physical activity, tobacco, or alcohol).	No comparator group
Outcome	Health behaviours i.e., one or more of dietary intake, physical activity/sedentary behaviour, smoking/tobacco use, alcohol use or anthropometric outcomes e.g., weight; functional capacity (i.e., 6-minute walk test); health care service use (length of stay); quality of life	Anthropometric or dietary outcomes where the intervention aimed to promote weight maintenance/gain (not weight loss)
Study design	Individually or cluster randomised controlled trials.	Articles not published in English

### Searches

We conducted a search of the following databases from inception to May 2021: MEDLINE, PubMed, PsychINFO, CINAHL, Web of Science, Google Scholar, Clinical Trials and Embase. The main search terms were: health behaviours AND trial AND surgery. S1 File details the search for MEDLINE. Reference lists of included trials were hand searched to check for any additional studies not identified by the main searches. An updated search was conducted in Medline from 2021 to August 2022 and again in March 2023. We only updated Medline due to the likelihood that most abstracts would be found there.

### Data extraction

Results were uploaded to Rayyan [34], a software platform used for screening, and duplicates were removed. Two independent reviewers screened study titles, abstracts and full texts (from among MF, MR, LE, KH, GC, MN, CG). If there were disagreements these were resolved by consensus or by a third reviewer (from among EK and JP). Full texts were uploaded to Covidence systematic review software [35]. All decisions of inclusion or exclusion were automatically recorded in Covidence, and reviewers were blinded to each other’s decisions. Data about study characteristics were extracted from among five authors (from among MF, MR, LM, CG, MN) (see S2 Table in S1 File) and three authors independently extracted outcome data. We contacted four authors [36–39] of included trials for further information about outcome data. For one trial the data were not accessible [39] and for one trial [37] the authors provided mean changes and standard deviations. The author of one study [40] was contacted to clarify intervention components. As no response was received this study was not included in this review.

### Outcomes, summary measures and synthesis of results

The aim of this review was to provide an overview of the effect of Prehabilitation interventions initiated before surgery that address health risk behaviours and was exploratory in nature. Therefore, there were several outcomes of interest all of which held equal value: healthcare usage (length of stay (LOS)); behavioural outcomes (diet, anthropometry, physical activity, smoking, alcohol use), functional outcomes (functional capacity i.e., 6 Minute Walk Test (6MWT)), and quality of life (QoL). In the narrative synthesis, only findings of definitive trials (i.e., not pilot/feasibility studies) were presented; these studies are more likely to be powered to detect between-group differences. Outcomes assessed after the prehabilitation intervention and just prior to surgery (herein referred to as the ‘pre-surgery’ timepoint) were extracted where reported. Post-surgery outcomes were only extracted if both groups received the same treatment after surgery i.e., both the intervention and control group did or did not receive rehabilitation after surgery. To examine the impact of interventions on health inequalities, we extracted data about income, ethnicity, employment, education, deprivation, and sex.

### Meta-analyses

Many studies reported LOS, 6MWT, QoL, BMI, and smoking behaviour and were synthesised in a meta-analysis. We took a pragmatic approach and identified the most common measurement for each outcome with a minimum of three studies. Post-surgery outcomes were assessed at multiple timepoints. As they are likely to change over time, we analysed outcomes at discrete timepoints (e.g., 4 weeks, 1-year) rather than analysing aggregated outcomes. All analyses were conducted using Review Manager 5.4 [41]. Random effects models were used as the diversity of intervention components and comparator conditions meant that treatment effects were expected to differ. Many studies did not report mean changes and, therefore, we checked there were no data to suggest that baseline measures differed between groups, and then entered follow-up data.

Trials that reported LOS as a mean (SD) were included in the meta-analysis and a pooled mean difference was calculated. Mean difference was calculated for 6MWT outcomes at pre-surgery and post-surgery. *I* ^2^ were reported to quantify heterogeneity and Tau^2^ to report between study variances. We generated funnel plots to evaluate small study effects (an indication of publication bias). We conducted the same analysis of QoL and BMI change at pre-surgery only, as there were not enough studies post-surgery. A pooled risk ratio was calculated for smoking cessation pre-operatively and at 12 months using random effects models. Three trials [42–44] measured smoking abstinence across pre-surgery and three weeks post-surgery and were included in the analysis as above.

#### Risk of bias

Two authors (CM and HG) independently assessed the risk of bias for each included study, using the Cochrane Risk of Bias Tool v2 (ROB2) [45]. For incomplete outcome data, a high risk of bias was defined as ≥20% attrition. We resolved disagreements by discussion or consulting a third review author (EK).

## Results

**Fig 1:**
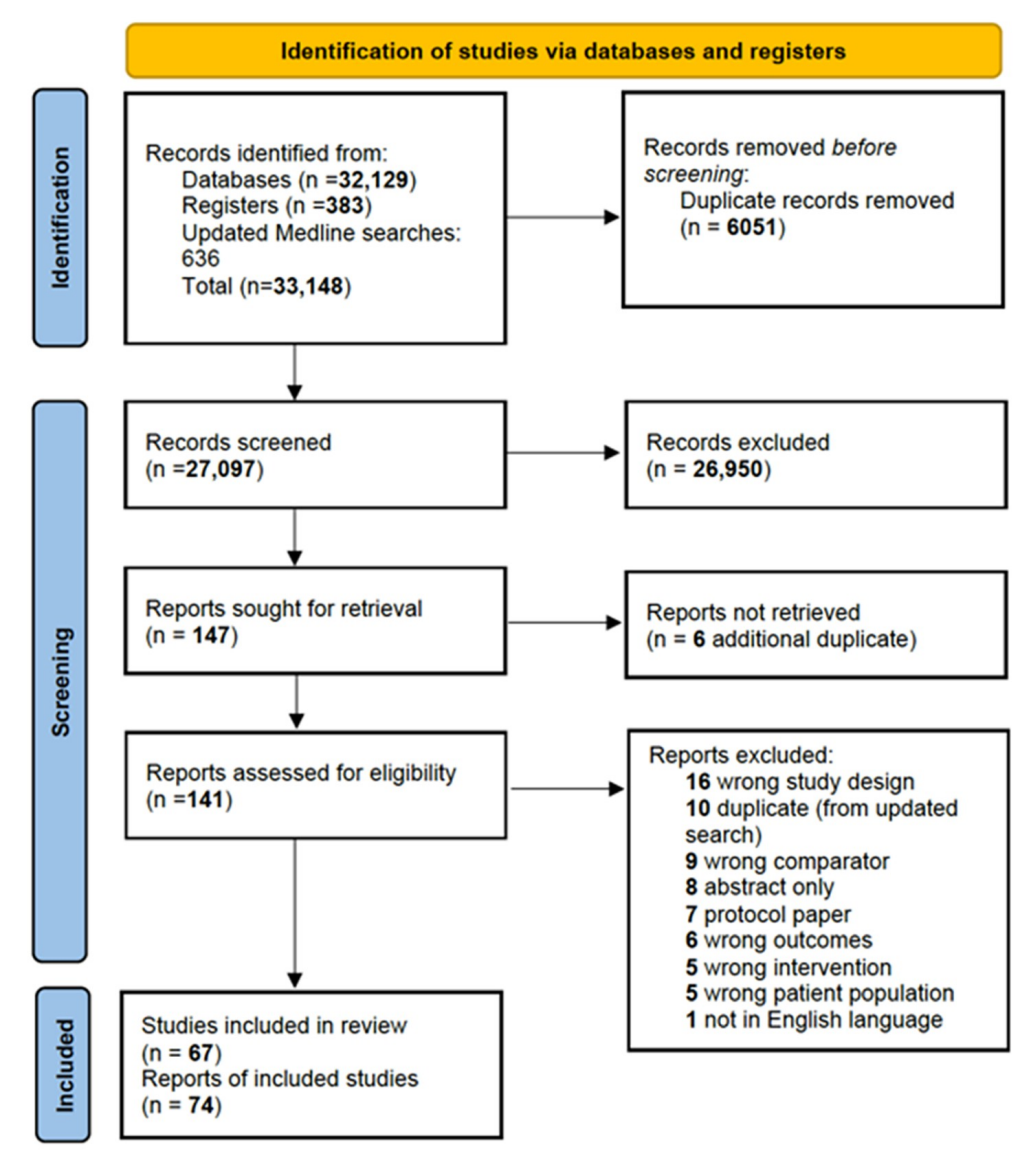
PRISMA flow diagram.

There were 67 unique trials that met our eligibility criteria. Some trials had published more than one paper and, therefore, the total number of papers that were eligible for inclusion was 74. **Fig 1** shows the PRISMA flow diagram.

### Study characteristics

Most of the trials (S2 Table in S1 File) were conducted in Canada (n = 15) [27,46–63], UK (n = 11) [64–74] and USA (n = 9) [39,75–83]. The number of participants included ranged from 3 to 761 (median = 88). Two trials included only women [44,82], two included only men [60,61,78] and two did not record sex [83,84]. On average the percentage of women was 42.3%, and the average age (of those that reported mean age) was 62.8 years. Only 13 studies reported a measure of socioeconomic status [37,43,46,59–61,70,78,79,85–89] and these included education, income and index of multiple deprivation (a UK measure). Only seven trials reported ethnicity [60,61,70,72,78,80,82,88]. The trial designs included RCTs (n = 49; 73.1%) [36–39,42–44,46–49,52,54–57,59,63–67,69,75–78,81,82,84–107], feasibility/pilot RCTs (n = 18; 26.9.%) [50,51,53,58,60–62,68,70–74,80,83,108–111], including one pilot/feasibility cluster RCT [79].

### Intervention characteristics

There were 18 (26.9%) interventions that targeted multiple behaviours [36,37,47–52,57,58,62,67,69,74,76,78,81,87,94,111] and 49 (73.1%) that focused on a single behaviour [38,39,42–44,46,53–56,59–61,64–66,68,70–73,75,77,79,80,82–86,88,90–93,95–110] amongst the behaviours of interest. Most interventions targeted physical activity (n = 34; 50.7%) [38,39,46,53,56,60,61,64–66,70,71,73,75,77,80,82–84,86,88,90–93,95–98,100–102,105,108–110], physical activity and dietary intake (n = 11; 16.4%) [37,47,52,57,58,62,67,74,76,78,81,87,94] and smoking behaviour (n = 13; 19.4%) [42–44,54,55,59,63,68,79,99,103,104,106,107]. One intervention focused solely on alcohol use [72] and one on dietary intake [85]. Four interventions focused on changing all four behaviours (physical activity, dietary intake, smoking and alcohol use) [36,48,69,111]. The duration of intervention ranged from one session to nine months, with a median duration of four weeks, although duration was not reported in 13 studies. In many cases, it was difficult to ascertain up to when the intervention was delivered in relation to surgery e.g., the intervention was delivered up to the day of surgery, or the intervention was completed a week prior to surgery. As a proxy we used the time the outcome data was collected pre-surgery. Intervention characteristics are presented in S3 Table in S1 File.

### Outcomes

There were 18 trials that did not specify when the pre-surgery outcome was measured other than stating ‘pre-surgery’. The other trials measured pre-surgery outcomes between one week before surgery, up to the day of admission for surgery. S1 and S2 Figs in S1 File comprehensively depict the behaviours targeted, the outcomes assessed and at which time point for all studies.

#### Length of stay

**Fig 2:**
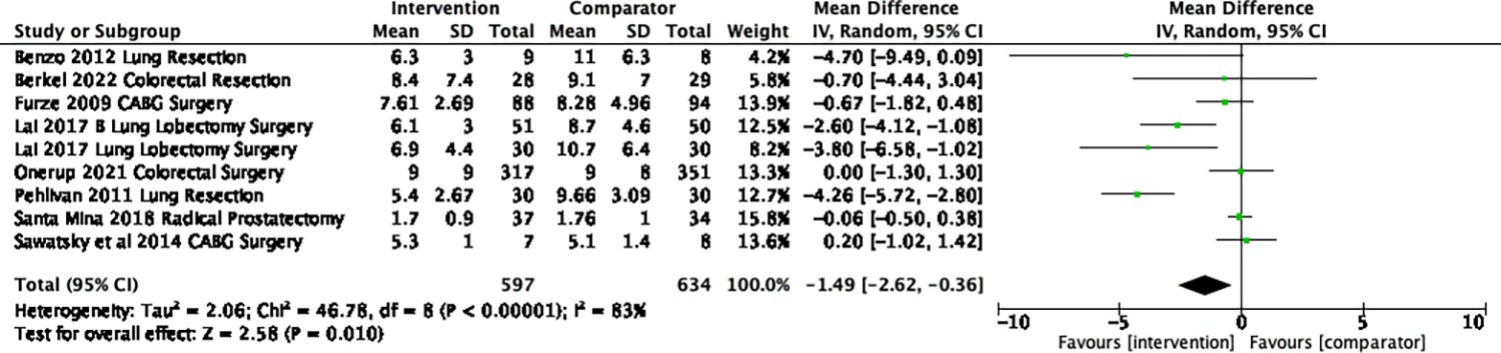
Mean difference in the length of stay (days).

There were 44 trials [36–39,42,44,46,48,49,51,54,56–62,64–67,70–73,75,77,81,84,88,91,92,94–97,99–102,104,108–110,112]) that reported LOS. Of the 32 trials that assessed between-group differences, seven trials (21.9%) [46,64,84,91,95,96,100] found that the Prehabilitation group had a shorter LOS than the comparator group. Only nine trials reported length of hospital stay as means (SD) and were included in a meta-analysis [60,62,66,88,92,95,96]. There was a mean difference of -1.5 days (95%CI –2.6 to -0.4, *I*^*2*^ 83%, Tau^2^ 2.06, p = 0.01) in favour of the intervention group (**Fig 2**). In a post-hoc sensitivity analysis focusing on the type of surgery, only lung cancer surgery (n = 4) was associated with a significant difference of -3.6 days (95% CI -4.5 to -2.6, p<0.001, *I*^*2*^ 0%). As there were less than two of each other surgical specialty we combined them and there was no difference in LOS (-0.1 days, 95% CI -0.5 to 0.3, p = 0.59, *I*^*2*^ 0%).

#### Functional capacity

**Fig 3:**
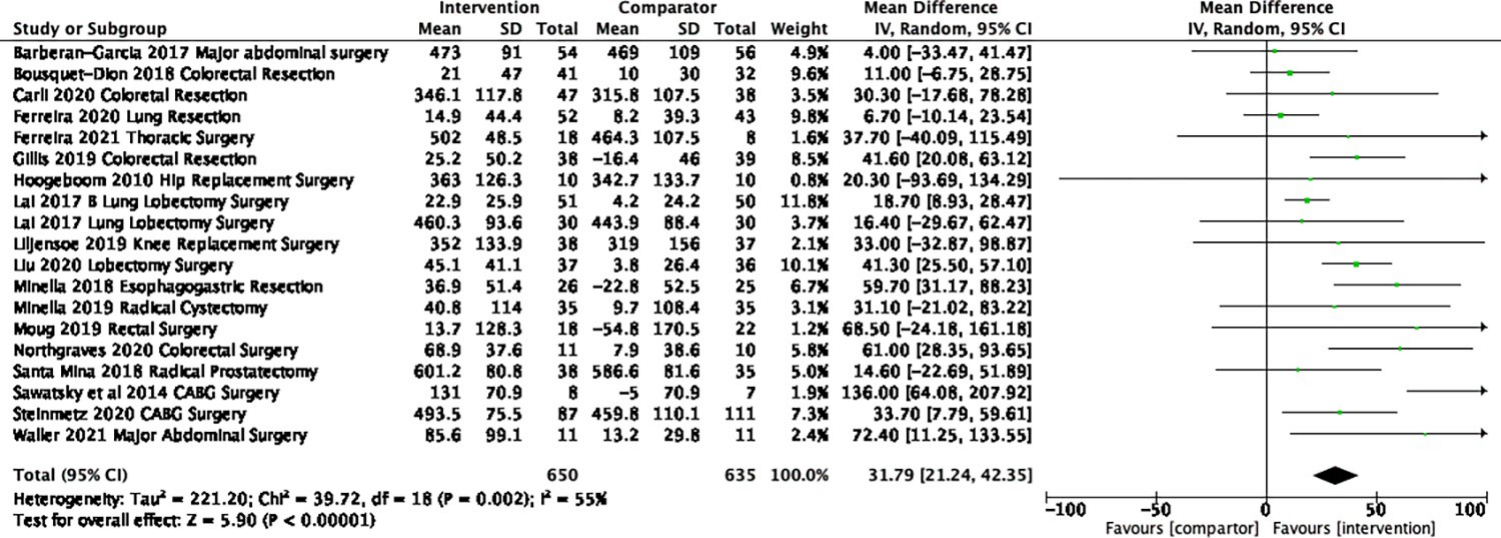
Mean difference in 6MWT from baseline to pre-surgery.

Thirty-one trials [36–38,47–49,52,53,56–58,60,62,70,71,74,80,83,85,86,91,93–97,102,105,108–110,112] assessed functional capacity, predominately using the 6MWT. There were 19 trials [37,47–50,57,58,60,62,70,71,74,85,91,94–96,105,108] (n = 1285) included in the meta-analyses of 6MWT pre surgery as they reported data that were able to be synthesized (**Fig 3**). A significant mean difference of 31.8m (95% CI 21.2 to 42.4, I^2^ 55% Tau^2^ 221.27 P<0.001) was found in favour of the intervention group. In a sensitivity analysis exploring only Prehabilitation interventions for lung cancer surgery and colorectal surgery there was no change in the overall results. The duration of the interventions ranged from one week to 16 weeks, with three trials not reporting duration and some reporting a range, thus limiting our ability to examine the duration of intervention on outcomes.

**Fig 4:**
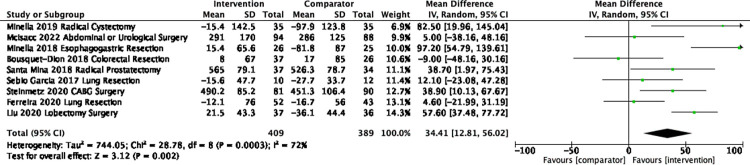
Mean difference in 6MWT from baseline to approximately 4 weeks post-surgery.

A meta-analysis of nine trials [37,47,49,56–58,60,102,105] (n = 798) assessing post-surgery 6MWT at four-week follow-up found a mean difference of 34.4m (95%CI 12.8 to 56.0, *I*^*2*^ 72% Tau2 744.05, P = 0.002) (**Fig 4**). In a sensitivity analysis, we removed the trial by Minnella et al. [57] with a follow-up ranging from 4–8 weeks and the results did not change. There were four trials (n = 305) in the meta-analysis at eight weeks follow-up and there was no longer a significant difference (15.8 m, 95% CI -8.8 to 40.3, *I*^*2*^ 62%, Tau^2^ 354.89) (S3 Fig in S1 File). The one trial [67] with follow-up up to 26-weeks after surgery found no between-group difference.

#### Quality of life

QoL was assessed in 34 trials [36,39,46,48–50,58,60,62,65,67,69–73,76–78,85–87,90,91,94–96,98,100–102,105,110,112], most commonly through the 36-Item Short Form Health Survey (SF-36). Of 21 trials comparing between-group QoL before surgery, two trials (9.5%) [69,105] found that QoL was significantly greater in the Prehabilitation group across all questionnaire subscales, while seven trials (33.3%) trials found mixed evidence varying by subscale and/or questionnaires [36,39,46,65,67,86]. Most trials that used SF-36 to measure QoL reported the outcomes by the physical component summary score (PCS) and mental component summary score (MCS). There were 11 trials [46,48,50,58,65,67,73,85,91,98,101] (n = 1167) included in the PCS meta-analysis and there were no significant differences between intervention and comparator groups before surgery (mean difference 1.1, 95% CI -0.07 to 2.4, I^2^ 30%, Tau 1.12, p = 0.07) (S4 Fig in S1 File). Twelve trials [46,48–50,58,65,67,73,85,91,98,101] (n = 1185) were included in the MCS and there were no significant differences between groups (mean difference 0.08, 95% CI –1.3 to 1.4, *I*^*2*^ 14%, Tau^2^ 0.74, p = 0.91) (S5 Fig in S1 File).

There were eight trials that measured quality of life using the SF-36 post surgery and time points varied from discharge of surgery to one year, thus, it was not possible to conduct a meta-analysis. Descriptively, within one month after surgery, one of seven trials [49] (14.3%) found a favourable effect of Prehabilitation among some questionnaire subscales and one trial (14.3%) found varying results by questionnaire scale [90]. Up to 12-weeks after surgery, one of eight trials [102] found a significant difference favouring Prehabilitation, while one of seven [49] found varying results. Up to 26 weeks after surgery, three of four trials found some evidence supporting Prehabilitation improving quality of life [39,46,90]. No trials with one-[87,98] or two-year [76] follow-up after surgery found between-group differences in QoL.

#### Anthropometry

Of the six trials assessing anthropometry [67,69,76,78,81,85], one of these [76] had only data at two years post-surgery. BMI was measured at pre-surgery and the mean difference was –0.9 kg/m^2^ (95% CI –1.82 to 0.01, p = 0.05, *I*^*2*^ 73%, Tau^2^ 0.58) in favour of the intervention group (S6 Fig in S1 File). Post-surgery, no between-group differences in anthropometry were observed at 2-year follow-up [76].

#### Smoking behaviour

**Fig 5:**
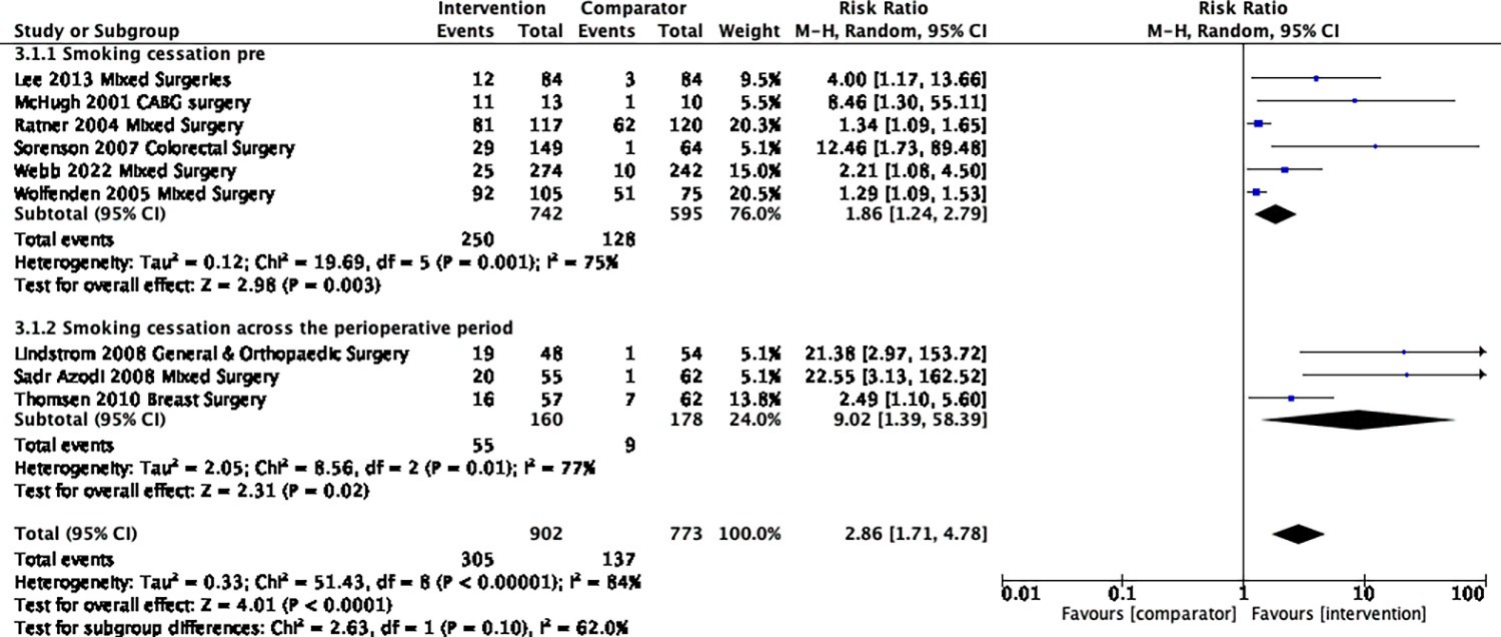
Risk Ratio of smoking cessation at pre-surgery and across the peri-operative period.

Smoking behaviour was assessed in 17 trials [42–44,54,55,59,63,67–69,79,89,99,103,104,106,107,111] through various self-report methods (e.g., questionnaires, interviews), with ten studies [42–44,54,59,63,103,104,106,107] using biochemical validation (e.g., exhaled CO, urinary cotinine) at least at one-time point in at least a proportion of the participant sample. There were nine trials (n = 1675; no feasibility/pilot studies) [42–44,54,59,69,89,103,107] included in the meta-analysis of smoking abstinence at pre-surgery and the risk ratio was 2.9 (95% CI 1.7 to 4.8, *I*^*2*^ 84%, Tau2 0.33, P <0.001) significantly in favour of the intervention group (**Fig 5**). Three trials [42–44] measured smoking abstinence across the perioperative period i.e., from pre-surgery up to three-weeks post-surgery; in subgroup analysis there were no significant differences.

**Fig 6:**
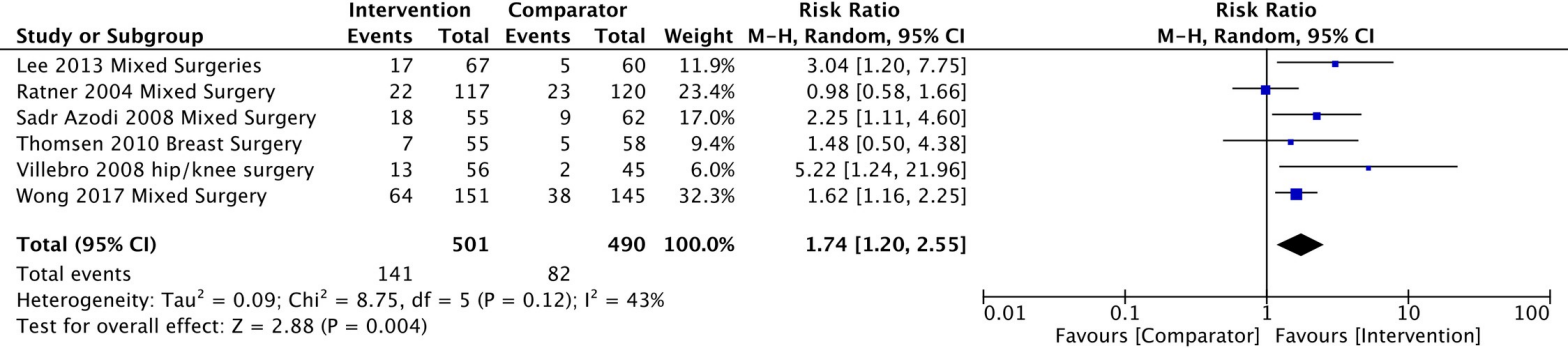
Risk ratio of smoking cessation at 12-months post-surgery.

There were six trials [43,44,55,59,63,106] (n = 991) included in the meta-analysis at 12 months for the abstinence outcome and the risk ratio was 1.74 (95% CI 1.20 to 2.55, I^2^ 43%, Tau^2^ 0.09, p = 0.004) (Fig 6).

#### Physical activity

Physical activity was assessed in 19 trials [47,48,50,52,58,60–62,69,70,74,78,82,86,87,90,91,94,98,108,110,111] through various objective (e.g. accelerometry), and subjective methods (e.g. self-report questionnaires and diaries). Of the eight trials assessing between-group differences pre-surgery, five (62.5%) trials [52,69,82,90,98] observed greater physical activity in the Prehabilitation group, while one trial [47] found that Prehabilitation benefited some components of physical activity (i.e., moderate to vigorous physical activity (MVPA)) but not others. Within one-month of surgery, one of six trials [16.7%] found significantly greater physical activity levels in the Prehabilitation group. Up to 12 weeks after surgery, one trial of three [47] found a significant group difference that favoured Prehabilitation, although this was only for MVPA [47]. One of two trials [90] assessing physical activity at 6-months post-surgery observed a between-group difference that favoured the Prehabilitation group. One [98] of two trials with 12-month follow-up after surgery found a significant effect favouring Prehabilitation

#### Dietary intake

Two trials assessed dietary intake [52,78]; one through a 2-day dietary recall [78], and the other through a 3-day recall [52]. The one trial that assessed between-group difference in intake before surgery [78] found that daily energy intake was significantly lower in the Prehabilitation group. Between-group difference in dietary intake post-surgery was not assessed in either study.

#### Alcohol use

Alcohol use was assessed in two studies, one via the AUDIT-C [72] and one via a bespoke questionnaire [111]. Both were pilot and/or feasibility trials and, therefore, between-group comparisons are not summarised here.

#### Health inequalities

None of the trials included in this review examined differential effects by socioeconomic characteristics, nor did any specifically target lower sociodemographic groups (e.g., lower income patients). Therefore, we were unable to examine the impact of behavioural Prehabilitation interventions on health inequalities.

#### Publication bias

There was no evidence of publication bias in the studies included in the meta-analyses (S7-S15 Figs in S1 File).

#### Risk of bias

**Fig 7:**
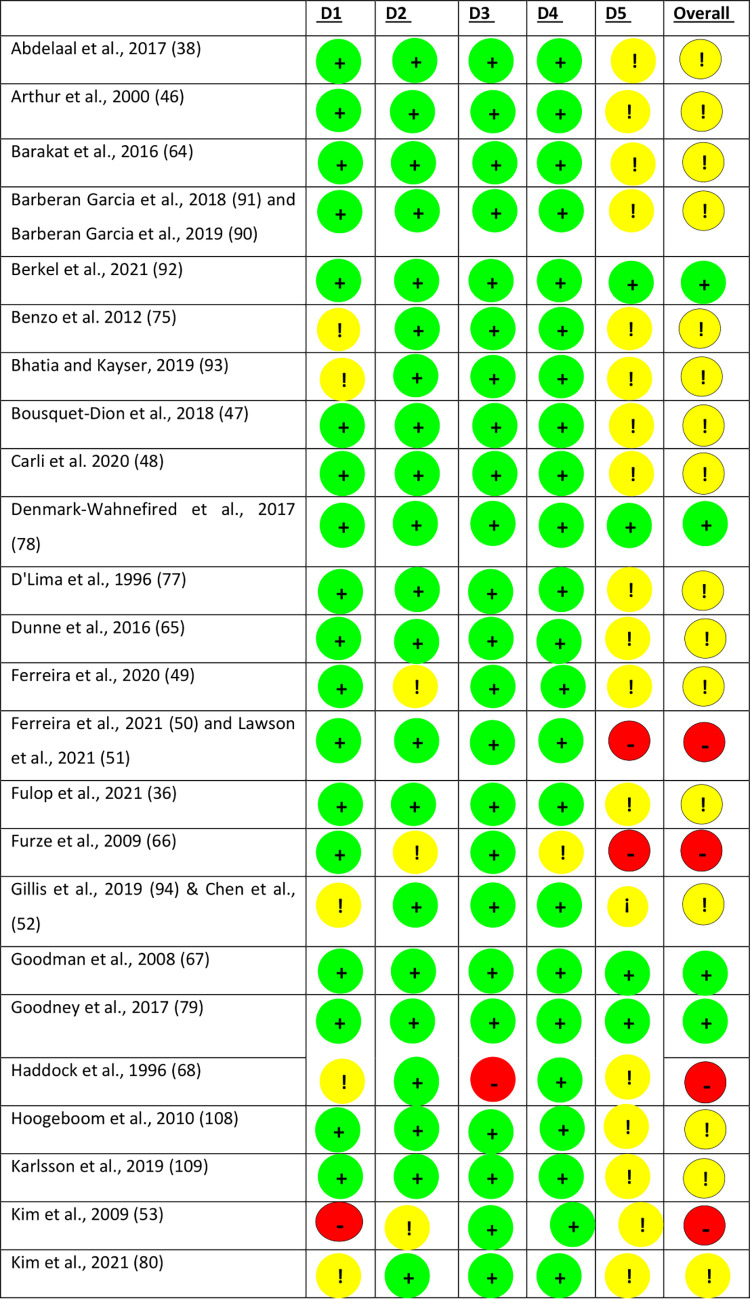
Risk of bias assessment for included studies.

There were eight trials that were considered high risk of bias, 48 as unclear risk of bias and 11 as low risk of bias (Fig 7).

## Discussion

Prehabilitation interventions were effective at optimizing functional capacity, and smoking cessation prior to surgery. These improvements may have contributed to an average shorter length of stay (-1.5 days) which was observed in the Prehabilitation group, but this is likely to be specific to Prehabilitation for lung cancer surgery as found in sensitivity analysis. There was no evidence that Prehabilitation interventions improved QoL, or reduced BMI just before surgery, although the BMI outcomes were only reported by a small number of trials (n = 4). Regarding post-surgery outcomes, improvements in physical function were sustained for up to four weeks post-surgery. Only smoking cessation data at 12 months could be quantitatively synthesised; greater rates of smoking cessation in the prehabilitation group were observed. That improvements in smoking outcomes were sustained at 12-months post-surgery suggests that the surgical encounter holds promise as a teachable moment for longer-term behavioural change. No studies reported outcomes by health inequality measures, therefore, we could not determine whether Prehabilitation interventions are equitable.

Prehabilitation reduced mean LOS by 1.5 days, similar to findings of another systematic review [113] which found a reduction of 1–2 days compared in patients undergoing joint surgery. However, another systematic review only found a reduction of -0.27 days in colon and rectal cancer patients [114]. In our sensitivity analysis we found that for patients receiving lung surgery LOS was reduced by 3.5 days. There were not more than two surgical specialties and therefore other specialties could not be compared. This suggests that the effect of Prehabilitation differs across surgical procedures and should be investigated in future research. Additionally, we found that Prehabilitation improved 6MWT both before surgery (32m) and after (38m) both exceeding the upper range of the minimal clinically important difference of 30.5 m [115]. Thus, Prehabilitation may improve fitness to undertake the surgery.

Prehabiliation improved rates of smoking cessation before surgery. Research suggests that at least four weeks of successful smoking cessation can reduce respiratory complications, while at least three to four weeks can reduce wound healing complications [116]. The duration of several smoking interventions included in this review was less than three weeks, and, therefore, their impact on intra- and peri-operative health outcomes may be limited. We found no difference in rates of smoking cessation at 12-months after surgery. This is similar to findings of a review whereby hospital initiated smoking cessation programmes (in admitted patients) increased smoking cessation for six to 12 months after discharge (risk ratio (RR) 1.37, 95% confidence interval (CI) 1.27 to 1.48; 25 trials) [117]. These effects were produced by high-intensity behavioural interventions that included at least one month of supportive contact after discharge. In times of integrated care, it assumed that community provision has a role to support successful longer-term cessation beyond hospital discharge. Indeed, referral to community smoking cessation services after discharge is a critical component of the evidence-based hospital-initiated tobacco dependency treatment services currently being implemented in the UK as part of the NHS Long Term Plan [118]. However, some studies included in the current review would not be considered high-intensity, and indeed, some consisted of ‘one-off’ counselling sessions e.g., one 15-minute session, suggesting that lower intensity interventions may still be effective at producing longer-term benefits.

We did not find an improvement in mental or physical QoL in the pre-surgery period. This may be because, while interventions produce significant improvements in some outcomes (functional capacity and smoking), they may not be substantial enough to produce a noticeable effect. For example, an improvement of 32 m in the 6MWT may be clinically significant but may not have a tangible impact on patients’ QoL. It may also be those with serious conditions who are experiencing pain and/or discomfort may only achieve improved QoL through surgery. Other reviews that have specifically investigated psychological prehabilitation interventions have found improvements in QoL [119]. Therefore, behavioural interventions alone may not be sufficient to improve QoL in the period leading up to surgery, and psychological support may be necessary.

A systematic review of lifestyle weight loss interventions found that weight loss of 7.2% before surgery in people with obesity can reduce hospital length of stay by 27% [120]. However, most studies included in this review were patients receiving bariatric surgery and these studies were excluded in our review. Intentional weight loss before surgery remains contentious. There are observations of a weight-outcome paradox whereby people with a BMI > 30 kg/m^2^ appear to experience better outcomes than those with lower BMIs specifically for cardiovascular surgery [121]. One proposed explanation is that plentiful reserves of fat provide energy during periods of accelerated catabolism after major surgery [122], reducing mortality risk. Another is that chronic inflammation, characteristic of obesity, pre-conditions the body against acute excessive inflammation [123], reducing mortality risk. More research is needed to understand under what circumstances (e.g., co-morbidities, baseline BMI, surgery type) weight loss is beneficial, what amount of weight loss is appropriate and at what stage of the surgical pathway.

### Strengths and limitations

This is the first systematic review to consolidate the literature on the characteristics and effectiveness of behavioural prehabilitation interventions targeting health risk behaviours of public health importance (i.e., physical activity, diet (and weight loss), alcohol use and smoking) for improving a range of important outcomes across surgical specialties. As we included unimodal and multimodal interventions, we could not identify which specific intervention components were most effective. While this review focused on four risk behaviours, some of the interventions included other components such as psychological support, protein supplementation and breathing exercises. This may have influenced outcomes, although, these trials were very few. Around a quarter of trials were either pilot/feasibility trials and only eight (11.9%) were judged to have a low risk of bias, thus overall, there is limited high quality evidence. The risk of bias may have been unclear or high because reporting of intervention and assessment timepoints were not clear; we suspect this may reflect uncertainty around surgery schedules and/or changing health circumstances. Lastly, due to heterogeneity in assessment reporting, some studies were not included in the meta-analyses.

### Unanswered questions and future research

There needs to be better agreement of outcome measures (to synthesise findings) and better reporting including descriptions of the interventions (e.g., use of the TIDiER checklist [124]) to enable service providers to identify the most effective service for their population. Also, the paucity of longer-term post-surgery data means it remains unknown whether Prehabilitation interventions can promote longer-term behavioural change and health improvements. The Prehabilitation literature tends to focus on physiological and mechanistic outcomes with little consideration for the role of behavioral science [125]. To truly leverage surgery as a teachable moment for longer-term outcomes, future interventions must draw on behavioral science [126]. As socioeconomic position is an independent predictor of surgical complications [127,128] examining the effect of Prehabilitation interventions in different socioeconomic groups is important for future research; routine service data may help answer this question rather than trials. Intervention effectiveness across socioeconomic groups is critical in future research investigating digital prehabilitation interventions given poorer digital access and literacy among disadvantaged persons and those with complex needs [129]. Most Prehabilitation interventions included exercise, hence why we found good evidence for improved physical function. However, there were very few studies that focused on alcohol use, dietary intake and weight loss and we did not explore adverse effects of prehabilitation interventions. The cost-efficiency and resource implications of Prehabilitation interventions are an important consideration for hospital systems and should be a priority for future research. If costs associated with delivery of the Prehabilitation service are less than costs saved through bed days released (approximately £342/bed/day in the UK [130]), the service would be ‘cost-efficient’ [131], and savings could be used to expand provision of Prehabilitation services.

## Conclusions

Behavioural Prehabilitation interventions could be offered to patients across different surgery specialties prior to surgery to help improve functional capacity and smoking cessation outcomes which may enable them to be discharged sooner; however, evidence for shorter length of stay was only observed for patients undergoing lung cancer surgery. That improvements in smoking outcomes were sustained at 12 months post-surgery suggests that the surgical encounter holds promise as a teachable moment for longer-term behavioural change. Given the paucity of data on the effects on other behavioural risk factors, more research grounded in behavioural science and with longer-term follow-up is needed to further investigate this potential.

## Supporting information

S1 File Contains all the supporting files, tables and figures. (DOCX)

## References

[1] Dobson GP. Trauma of major surgery: A global problem that is not going away. Int J Surg. 2020;81:47–54. doi: 10.1016/j.ijsu.2020.07.017 32738546 PMC7388795

[2] Desborough JP. The stress response to trauma and surgery. Br J Anaesth. 2000;85(1):109–17. doi: 10.1093/bja/85.1.109 10927999

[3] Ghaferi AA, Birkmeyer JD, Dimick JB. Variation in Hospital Mortality Associated with Inpatient Surgery. New England Journal of Medicine. 2009;361(14):1368–75. doi: 10.1056/NEJMsa0903048 19797283

[4] Organisation for Economic Co-operation and Development (OECD). Waiting times 2020 [Available from: https://www.oecd.org/els/health-systems/waiting-times.htm.

[5] NHS England. Delivery plan for tackling the COVID-19 backlog of elective care. 2022. 10.1136/bmj.o995 35440437

[6] Wynter-Blyth V, Moorthy K. Prehabilitation: preparing patients for surgery. BMJ. 2017;358:j3702. doi: 10.1136/bmj.j3702 28790033

[7] McBride CM, Emmons KM, Lipkus IM. Understanding the potential of teachable moments: the case of smoking cessation. Health Educ Res. 2003;18(2):156–70. doi: 10.1093/her/18.2.156 12729175

[8] van Stijn MFM, Korkic-Halilovic I, Bakker MSM, van der Ploeg T, van Leeuwen PAM, Houdijk APJ. Preoperative Nutrition Status and Postoperative Outcome in Elderly General Surgery Patients. Journal of Parenteral and Enteral Nutrition. 2013;37(1):37–43. 22549764 10.1177/0148607112445900

[9] Sugawara K, Yamashita H, Urabe M, Okumura Y, Yagi K, Aikou S, et al. Poor nutritional status and sarcopenia influences survival outcomes in gastric carcinoma patients undergoing radical surgery. European Journal of Surgical Oncology. 2020;46(10, Part A):1963–70. doi: 10.1016/j.ejso.2020.04.044 32402508

[10] Mullen JT, Moorman DW, Davenport DL. The obesity paradox: body mass index and outcomes in patients undergoing nonbariatric general surgery. Ann Surg. 2009;250(1):166–72. doi: 10.1097/SLA.0b013e3181ad8935 19561456

[11] Turan A, Mascha EJ, Roberman D, Turner PL, You J, Kurz A, et al. Smoking and perioperative outcomes. Anesthesiology. 2011;114(4):837–46. doi: 10.1097/ALN.0b013e318210f560 21372682

[12] Warner DO. Tobacco dependence in surgical patients. Curr Opin Anaesthesiol. 2007;20(3):279–83. doi: 10.1097/ACO.0b013e3280c60c3b 17479035

[13] Warner DO. Perioperative abstinence from cigarettes: physiologic and clinical consequences. Anesthesiology. 2006;104(2):356–67. doi: 10.1097/00000542-200602000-00023 16436857

[14] Nath B, Li Y, Carroll JE, Szabo G, Tseng JF, Shah SA. Alcohol exposure as a risk factor for adverse outcomes in elective surgery. J Gastrointest Surg. 2010;14(11):1732–41. doi: 10.1007/s11605-010-1350-4 20839071

[15] Felding C, Jensen LM, Tønnesen H. Influence of alcohol intake on postoperative morbidity after hysterectomy. Am J Obstet Gynecol. 1992;166(2):667–70. doi: 10.1016/0002-9378(92)91695-7 1536250

[16] Tonnesen H, Kehlet H. Preoperative alcoholism and postoperative morbidity. Br J Surg. 1999;86(7):869–74. doi: 10.1046/j.1365-2168.1999.01181.x 10417555

[17] Warner DO. Surgery as a Teachable Moment: Lost Opportunities to Improve Public Health. Archives of Surgery. 2009;144(12):1106–7. doi: 10.1001/archsurg.2009.205 20026826

[18] McDonald S, Yates D, Durrand JW, Kothmann E, Sniehotta FF, Habgood A, et al. Exploring patient attitudes to behaviour change before surgery to reduce peri-operative risk: preferences for short- vs. long-term behaviour change. Anaesthesia. 2019;74(12):1580–8. doi: 10.1111/anae.14826 31637700

[19] ECDA EatNa. Towards an EU strategic framework for the prevention of NCDs. 2019.

[20] World Health Organization. Preventing chronic diseases: a vital investment. WHO global report. Geneva: World Health Organization; 2005.

[21] Cancer Research UK. Surgery for cancer 2022 [Available from: https://www.cancerresearchuk.org/about-cancer/cancer-in-general/treatment/surgery/about.

[22] Nuffield Trust. Elective (planned) treatment waiting times. 2022.

[23] Levy N, Selwyn DA, Lobo DN. Turning ‘waiting lists’ for elective surgery into ‘preparation lists’. British Journal of Anaesthesia. 2020;126(1):1–5. 32900503 10.1016/j.bja.2020.08.021

[24] Sommer I, Griebler U, Mahlknecht P, Thaler K, Bouskill K, Gartlehner G, et al. Socioeconomic inequalities in non-communicable diseases and their risk factors: an overview of systematic reviews. BMC Public Health. 2015;15(1):914. doi: 10.1186/s12889-015-2227-y 26385563 PMC4575459

[25] Marmot M, Bell R. Social determinants and non-communicable diseases: time for integrated action. BMJ. 2019;364:l251. doi: 10.1136/bmj.l251 30692093 PMC6348404

[26] McIsaac DI, Gill M, Boland L, Hutton B, Branje K, Shaw J, et al. Prehabilitation in adult patients undergoing surgery: an umbrella review of systematic reviews. Br J Anaesth. 2022;128(2):244–57. doi: 10.1016/j.bja.2021.11.014 34922735

[27] Gillis C, Buhler K, Bresee L, Carli F, Gramlich L, Culos-Reed N, et al. Effects of nutritional prehabilitation, with and without exercise, on outcomes of patients who undergo colorectal surgery: a systematic review and meta-analysis. Gastroenterology. 2018;155(2):391–410. e4. 29750973 10.1053/j.gastro.2018.05.012

[28] Luther A, Gabriel J, Watson RP, Francis NK. The impact of total body prehabilitation on post-operative outcomes after major abdominal surgery: a systematic review. World journal of surgery. 2018;42(9):2781–91. doi: 10.1007/s00268-018-4569-y 29546448

[29] Waterland JL, McCourt O, Edbrooke L, Granger CL, Ismail H, Riedel B, et al. Efficacy of prehabilitation including exercise on postoperative outcomes following abdominal cancer surgery: a systematic review and meta-analysis. Frontiers in surgery. 2021;8:628848. doi: 10.3389/fsurg.2021.628848 33816546 PMC8017317

[30] Åsberg K, Bendtsen M. Perioperative digital behaviour change interventions for reducing alcohol consumption, improving dietary intake, increasing physical activity and smoking cessation: a scoping review. Perioperative Medicine. 2021;10(1):18. doi: 10.1186/s13741-021-00189-1 34225795 PMC8258960

[31] Moher D, Liberati A, Tetzlaff J, Altman DG. Preferred reporting items for systematic reviews and meta-analyses: the PRISMA statement. PLoS Med. 2009;6(7):e1000097. doi: 10.1371/journal.pmed.1000097 19621072 PMC2707599

[32] Page MJ, McKenzie JE, Bossuyt PM, Boutron I, Hoffmann TC, Mulrow CD, et al. The PRISMA 2020 statement: an updated guideline for reporting systematic reviews. BMJ. 2021;372:n71. doi: 10.1136/bmj.n71 33782057 PMC8005924

[33] Caspersen CJ, Powell KE, Christenson GM. Physical activity, exercise, and physical fitness: definitions and distinctions for health-related research. Public Health Rep. 1985;100(2):126–31. 3920711 PMC1424733

[34] Ouzzani M, Hammady H, Fedorowicz Z, Elmagarmid A. Rayyan—a web and mobile app for systematic reviews. Systematoic reviews. 2016;5(210). doi: 10.1186/s13643-016-0384-4 27919275 PMC5139140

[35] Veritas Health Innovation. Covidence. Melbourne, Australia.

[36] Fulop A, Lakatos L, Susztak N, Szijarto A, Banky B. The effect of trimodal prehabilitation on the physical and psychological health of patients undergoing colorectal surgery: a randomised clinical trial. Anaesthesia. 2021;76(1):82–90. doi: 10.1111/anae.15215 32761611

[37] Liu Z, Qiu T, Pei L, Zhang Y, Xu L, Cui Y, et al. Two-Week Multimodal Prehabilitation Program Improves Perioperative Functional Capability in Patients Undergoing Thoracoscopic Lobectomy for Lung Cancer: A Randomized Controlled Trial. Anesth Analg. 2020;131(3):840–9. doi: 10.1213/ANE.0000000000004342 31348053

[38] Abdelaal GA, Eldahdouh SS, Abdelsamie M, Labeeb A. Effect of preoperative physical and respiratory therapy on postoperative pulmonary functions and complications after laparoscopic upper abdominal surgery in obese patients. Egyptian Journal of Chest Diseases and Tuberculosis. 2017;66(4):735–8.

[39] Rooks DS, Huang J, Bierbaum BE, Bolus SA, Rubano J, Connolly CE, et al. Effect of preoperative exercise on measures of functional status in men and women undergoing total hip and knee arthroplasty. Arthritis Rheum. 2006;55(5):700–8. doi: 10.1002/art.22223 17013852

[40] Crotty M, Prendergast J, Battersby MW, Rowett D, Graves SE, Leach G, et al. Self-management and peer support among people with arthritis on a hospital joint replacement waiting list: a randomised controlled trial. Osteoarthritis Cartilage. 2009;17(11):1428–33. doi: 10.1016/j.joca.2009.05.010 19486959

[41] Collaboration TC. Review Manager (RevMan). In: Centre TNC, editor. 5.4 ed: Copenhagen; 2014. p. Computer program.

[42] Lindström D, Sadr Azodi O, Wladis A, Tønnesen H, Linder S, Nåsell H, et al. Effects of a perioperative smoking cessation intervention on postoperative complications: a randomized trial. Ann Surg. 2008;248(5):739–45. doi: 10.1097/SLA.0b013e3181889d0d 18948800

[43] Sadr Azodi O, Lindström D, Adami J, Tønnesen H, Nåsell H, Gilljam H, et al. The efficacy of a smoking cessation programme in patients undergoing elective surgery: a randomised clinical trial. Anaesthesia. 2009;64(3):259–65. doi: 10.1111/j.1365-2044.2008.05758.x 19302637

[44] Thomsen T, Tønnesen H, Okholm M, Kroman N, Maibom A, Sauerberg ML, et al. Brief smoking cessation intervention in relation to breast cancer surgery: a randomized controlled trial. Nicotine Tob Res. 2010;12(11):1118–24. doi: 10.1093/ntr/ntq158 20855414

[45] Sterne JAC, Savović J, Page MJ, Elbers RG, Blencowe NS, Boutron I, et al. RoB 2: a revised tool for assessing risk of bias in randomised trials. Bmj. 2019;366:l4898. doi: 10.1136/bmj.l4898 31462531

[46] Arthur HM, Daniels C, McKelvie R, Hirsh J, Rush B. Effect of a preoperative intervention on preoperative and postoperative outcomes in low-risk patients awaiting elective coronary artery bypass graft surgery. A randomized, controlled trial. Ann Intern Med. 2000;133(4):253–62. doi: 10.7326/0003-4819-133-4-200008150-00007 10929164

[47] Bousquet-Dion G, Awasthi R, Loiselle S, Minnella EM, Agnihotram RV, Bergdahl A, et al. Evaluation of supervised multimodal prehabilitation programme in cancer patients undergoing colorectal resection: a randomized control trial. Acta Oncol. 2018;57(6):849–59. doi: 10.1080/0284186X.2017.1423180 29327644

[48] Carli F, Bousquet-Dion G, Awasthi R, Elsherbini N, Liberman S, Boutros M, et al. Effect of Multimodal Prehabilitation vs Postoperative Rehabilitation on 30-Day Postoperative Complications for Frail Patients Undergoing Resection of Colorectal Cancer: A Randomized Clinical Trial. JAMA Surg. 2020;155(3):233–42. doi: 10.1001/jamasurg.2019.5474 31968063 PMC6990653

[49] Ferreira V, Minnella EM, Awasthi R, Gamsa A, Ferri L, Mulder D, et al. Multimodal Prehabilitation for Lung Cancer Surgery: A Randomized Controlled Trial. Ann Thorac Surg. 2020;112(5):1600–8. doi: 10.1016/j.athoracsur.2020.11.022 33321089

[50] Ferreira V, Lawson C, Carli F, Scheede-Bergdahl C, Chevalier S. Feasibility of a novel mixed-nutrient supplement in a multimodal prehabilitation intervention for lung cancer patients awaiting surgery: A randomized controlled pilot trial. Int J Surg. 2021;93:106079. doi: 10.1016/j.ijsu.2021.106079 34464752

[51] Lawson C, Ferreira V, Carli F, Chevalier S. Effects of multimodal prehabilitation on muscle size, myosteatosis, and dietary intake of surgical patients with lung cancer—a randomized feasibility study. Appl Physiol Nutr Metab. 2021;46(11):1407–16. doi: 10.1139/apnm-2021-0249 34265218

[52] Chen BP, Awasthi R, Sweet SN, Minnella EM, Bergdahl A, Santa Mina D, et al. Four-week prehabilitation program is sufficient to modify exercise behaviors and improve preoperative functional walking capacity in patients with colorectal cancer. Support Care Cancer. 2017;25(1):33–40. doi: 10.1007/s00520-016-3379-8 27539131

[53] Kim DJ, Mayo NE, Carli F, Montgomery DL, Zavorsky GS. Responsive measures to prehabilitation in patients undergoing bowel resection surgery. Tohoku J Exp Med. 2009;217(2):109–15. doi: 10.1620/tjem.217.109 19212103

[54] Lee SM, Landry J, Jones PM, Buhrmann O, Morley-Forster P. The effectiveness of a perioperative smoking cessation program: a randomized clinical trial. Anesth Analg. 2013;117(3):605–13. doi: 10.1213/ANE.0b013e318298a6b0 23868890

[55] Lee SM, Landry J, Jones PM, Buhrmann O, Morley-Forster P. Long-term quit rates after a perioperative smoking cessation randomized controlled trial. Anesth Analg. 2015;120(3):582–7. doi: 10.1213/ANE.0000000000000555 25695576

[56] McIsaac DI, Hladkowicz E, Bryson GL, Forster AJ, Gagne S, Huang A, et al. Home-based prehabilitation with exercise to improve postoperative recovery for older adults with frailty having cancer surgery: the PREHAB randomised clinical trial. Br J Anaesth. 2022;129(1):41–8. doi: 10.1016/j.bja.2022.04.006 35589429

[57] Minnella EM, Awasthi R, Loiselle SE, Agnihotram RV, Ferri LE, Carli F. Effect of Exercise and Nutrition Prehabilitation on Functional Capacity in Esophagogastric Cancer Surgery: A Randomized Clinical Trial. JAMA Surg. 2018;153(12):1081–9. doi: 10.1001/jamasurg.2018.1645 30193337 PMC6583009

[58] Minnella EM, Awasthi R, Bousquet-Dion G, Ferreira V, Austin B, Audi C, et al. Multimodal Prehabilitation to Enhance Functional Capacity Following Radical Cystectomy: A Randomized Controlled Trial. Eur Urol Focus. 2019;7(1):132–8. doi: 10.1016/j.euf.2019.05.016 31186173

[59] Ratner PA, Johnson JL, Richardson CG, Bottorff JL, Moffat B, Mackay M, et al. Efficacy of a smoking-cessation intervention for elective-surgical patients. Res Nurs Health. 2004;27(3):148–61. doi: 10.1002/nur.20017 15141368

[60] Santa Mina D, Hilton WJ, Matthew AG, Awasthi R, Bousquet-Dion G, Alibhai SMH, et al. Prehabilitation for radical prostatectomy: A multicentre randomized controlled trial. Surg Oncol. 2018;27(2):289–98. doi: 10.1016/j.suronc.2018.05.010 29937184

[61] Au D, Matthew AG, Lopez P, Hilton WJ, Awasthi R, Bousquet-Dion G, et al. Prehabilitation and acute postoperative physical activity in patients undergoing radical prostatectomy: a secondary analysis from an RCT. Sports Med Open. 2019;5(1):18. doi: 10.1186/s40798-019-0191-2 31119491 PMC6531507

[62] Sawatzky JA, Kehler DS, Ready AE, Lerner N, Boreskie S, Lamont D, et al. Prehabilitation program for elective coronary artery bypass graft surgery patients: a pilot randomized controlled study. Clin Rehabil. 2014;28(7):648–57. doi: 10.1177/0269215513516475 24459173

[63] Wong J, Abrishami A, Riazi S, Siddiqui N, You-Ten E, Korman J, et al. A Perioperative Smoking Cessation Intervention With Varenicline, Counseling, and Fax Referral to a Telephone Quitline Versus a Brief Intervention: A Randomized Controlled Trial. Anesth Analg. 2017;125(2):571–9. doi: 10.1213/ANE.0000000000001894 28319515

[64] Barakat HM, Shahin Y, Khan JA, McCollum PT, Chetter IC. Preoperative Supervised Exercise Improves Outcomes After Elective Abdominal Aortic Aneurysm Repair: A Randomized Controlled Trial. Ann Surg. 2016;264(1):47–53. doi: 10.1097/SLA.0000000000001609 26756766

[65] Dunne DF, Jack S, Jones RP, Jones L, Lythgoe DT, Malik HZ, et al. Randomized clinical trial of prehabilitation before planned liver resection. Br J Surg. 2016;103(5):504–12. doi: 10.1002/bjs.10096 26864728

[66] Furze G, Dumville JC, Miles JN, Irvine K, Thompson DR, Lewin RJ. "Prehabilitation" prior to CABG surgery improves physical functioning and depression. Int J Cardiol. 2009;132(1):51–8. doi: 10.1016/j.ijcard.2008.06.001 18703241 PMC2643012

[67] Goodman H, Parsons A, Davison J, Preedy M, Peters E, Shuldham C, et al. A randomised controlled trial to evaluate a nurse-led programme of support and lifestyle management for patients awaiting cardiac surgery ’Fit for surgery: Fit for life’ study. Eur J Cardiovasc Nurs. 2008;7(3):189–95. doi: 10.1016/j.ejcnurse.2007.11.001 18160344

[68] Haddock J, Burrows C. The role of the nurse in health promotion: an evaluation of a smoking cessation programme in surgical pre-admission clinics. J Adv Nurs. 1997;26(6):1098–110. 9429959

[69] McHugh F, Lindsay GM, Hanlon P, Hutton I, Brown MR, Morrison C, et al. Nurse led shared care for patients on the waiting list for coronary artery bypass surgery: a randomised controlled trial. Heart. 2001;86(3):317–23. doi: 10.1136/heart.86.3.317 11514487 PMC1729900

[70] Moug SJ, Mutrie N, Barry SJE, Mackay G, Steele RJC, Boachie C, et al. Prehabilitation is feasible in patients with rectal cancer undergoing neoadjuvant chemoradiotherapy and may minimize physical deterioration: results from the REx trial. Colorectal Dis. 2019;21(5):548–62. doi: 10.1111/codi.14560 30657249

[71] Northgraves MJ, Arunachalam L, Madden LA, Marshall P, Hartley JE, MacFie J, et al. Feasibility of a novel exercise prehabilitation programme in patients scheduled for elective colorectal surgery: a feasibility randomised controlled trial. Support Care Cancer. 2020;28(7):3197–206. doi: 10.1007/s00520-019-05098-0 31712950 PMC7256075

[72] Snowden C, Lynch E, Avery L, Haighton C, Howel D, Mamasoula V, et al. Preoperative behavioural intervention to reduce drinking before elective orthopaedic surgery: the PRE-OP BIRDS feasibility RCT. Health Technol Assess. 2020;24(12):1–176. doi: 10.3310/hta24120 32131964 PMC7086307

[73] Tew GA, Batterham AM, Colling K, Gray J, Kerr K, Kothmann E, et al. Randomized feasibility trial of high-intensity interval training before elective abdominal aortic aneurysm repair. Br J Surg. 2017;104(13):1791–801. doi: 10.1002/bjs.10669 28990651

[74] Waller E, Rahman S, Sutton P, Allen J, Saxton J, Aziz O. Randomised controlled trial of patients undergoing prehabilitation with wearables versus standard of care before major abdominal cancer surgery (Trial Registration: NCT04047524). Colorectal disease. 2020;22(SUPPL 1):7‐. 10.1007/s00464-021-08365-6 PMC8758615 33723969

[75] Benzo R, Wigle D, Novotny P, Wetzstein M, Nichols F, Shen RK, et al. Preoperative pulmonary rehabilitation before lung cancer resection: results from two randomized studies. Lung Cancer. 2011;74(3):441–5. doi: 10.1016/j.lungcan.2011.05.011 21663994 PMC3191236

[76] Bernardi K, Olavarria OA, Dhanani NH, Lyons N, Holihan JL, Cherla DV, et al. Two-year Outcomes of Prehabilitation Among Obese Patients With Ventral Hernias: A Randomized Controlled Trial (NCT02365194). Ann Surg. 2020;275(2):288–94. 10.1097/SLA.0000000000004486 33201119

[77] D’Lima DD, Colwell CW Jr., Morris BA, Hardwick ME, Kozin F. The effect of preoperative exercise on total knee replacement outcomes. Clin Orthop Relat Res. 1996(326):174–82. doi: 10.1097/00003086-199605000-00020 8620638

[78] Demark-Wahnefried W, Rais-Bahrami S, Desmond RA, Gordetsky JB, Hunter GR, Yang ES, et al. Presurgical weight loss affects tumour traits and circulating biomarkers in men with prostate cancer. Br J Cancer. 2017;117(9):1303–13. doi: 10.1038/bjc.2017.303 28881355 PMC5672928

[79] Goodney PP, Spangler EL, Newhall K, Brooke BS, Schanzer A, Tan TW, et al. Feasibility and pilot efficacy of a brief smoking cessation intervention delivered by vascular surgeons in the Vascular Physician Offer and Report (VAPOR) Trial. J Vasc Surg. 2017;65(4):1152–60.e2. doi: 10.1016/j.jvs.2016.10.121 28190719

[80] Kim S, Hsu F-C, Groban L, Williamson J, Messier S. A pilot study of aquatic prehabilitation in adults with knee osteoarthritis undergoing total knee arthroplasty–short term outcome. BMC Musculoskeletal Disorders. 2021;22(1):388. doi: 10.1186/s12891-021-04253-1 33902505 PMC8074697

[81] Liang MK, Bernardi K, Holihan JL, Cherla DV, Escamilla R, Lew DF, et al. Modifying Risks in Ventral Hernia Patients With Prehabilitation: A Randomized Controlled Trial. Ann Surg. 2018;268(4):674–80. doi: 10.1097/SLA.0000000000002961 30048306

[82] Ligibel JA, Giobbie-Hurder A, Shockro L, Rhei E, Troyan S, Dominici LS, et al. Impact of a pre-operative exercise intervention on Ki-67 and metabolic markers in women with early breast cancer. Journal of Clinical Oncology. 2016;34(15_suppl):564–.

[83] Rengel KF, Mehdiratta N, Vanston SW, Archer KR, Jackson JC, Thompson JL, et al. A randomised pilot trial of combined cognitive and physical exercise prehabilitation to improve outcomes in surgical patients. Br J Anaesth. 2021;126(2):e55–e7. doi: 10.1016/j.bja.2020.11.004 33317805 PMC8040115

[84] Pehlivan E, Turna A, Gurses A, Gurses HN. The effects of preoperative short-term intense physical therapy in lung cancer patients: a randomized controlled trial. Ann Thorac Cardiovasc Surg. 2011;17(5):461–8. doi: 10.5761/atcs.oa.11.01663 21881371

[85] Liljensøe A, Laursen JO, H. Bliddal KS, Mechlenburg I. Weight loss intervention before total knee replacement:A 12-Month Randomized Controlled Trial. Scandinavian Journal of Surgery. 2019;Vol. 110(1) 3–12. doi: 10.1177/1457496919883812 31679465

[86] Lotzke H, Brisby H, Gutke A, Hägg O, Jakobsson M, Smeets R, et al. A Person-Centered Prehabilitation Program Based on Cognitive-Behavioral Physical Therapy for Patients Scheduled for Lumbar Fusion Surgery: A Randomized Controlled Trial. Phys Ther. 2019;99(8):1069–88. doi: 10.1093/ptj/pzz020 30951604 PMC6665875

[87] Nguyen C, Boutron I, Roren A, Anract P, Beaudreuil J, Biau D, et al. Effect of Prehabilitation Before Total Knee Replacement for Knee Osteoarthritis on Functional Outcomes: A Randomized Clinical Trial. JAMA Netw Open. 2022;5(3):e221462. doi: 10.1001/jamanetworkopen.2022.1462 35262716 PMC8908069

[88] Onerup A, Andersson J, Angenete A, Bock D, Börjesson M, Ehrencrona C, et al. Effect of short-term homebased pre- and postoperative exercise on recovery after colorectal cancer surgery (PHYSSURG-C): A randomized clinical trial. Annals of Surgery. 2021. 10.1097/SLA.0000000000004901 PMC8820776 33843798

[89] Wolfenden L, Wiggers J, Knight J, Campbell E, Rissel C, Kerridge R, et al. A programme for reducing smoking in pre-operative surgical patients: randomised controlled trial. Anaesthesia. 2005;60(2):172–9. doi: 10.1111/j.1365-2044.2004.04070.x 15644016

[90] Barberan-Garcia A, Ubre M, Pascual-Argente N, Risco R, Faner J, Balust J, et al. Post-discharge impact and cost-consequence analysis of prehabilitation in high-risk patients undergoing major abdominal surgery: secondary results from a randomised controlled trial. Br J Anaesth. 2019;123(4):450–6. doi: 10.1016/j.bja.2019.05.032 31248644

[91] Barberan-Garcia A, Ubré M, Roca J, Lacy AM, Burgos F, Risco R, et al. Personalised Prehabilitation in High-risk Patients Undergoing Elective Major Abdominal Surgery: A Randomized Blinded Controlled Trial. Ann Surg. 2018;267(1):50–6. doi: 10.1097/SLA.0000000000002293 28489682

[92] Berkel AEM, Bongers BC, Kotte H, Weltevreden P, de Jongh FHC, Eijsvogel MMM, et al. Effects of Community-based Exercise Prehabilitation for Patients Scheduled for Colorectal Surgery With High Risk for Postoperative Complications: Results of a Randomized Clinical Trial. Ann Surg. 2022;275(2):e299–e306. doi: 10.1097/SLA.0000000000004702 33443905 PMC8746915

[93] Bhatia C, Kayser B. Preoperative high-intensity interval training is effective and safe in deconditioned patients with lung cancer: A randomized clinical trial. J Rehabil Med. 2019;51(9):712–8. doi: 10.2340/16501977-2592 31468059

[94] Gillis C, Fenton TR, Sajobi TT, Minnella EM, Awasthi R, Loiselle S, et al. Trimodal prehabilitation for colorectal surgery attenuates post-surgical losses in lean body mass: A pooled analysis of randomized controlled trials. Clin Nutr. 2019;38(3):1053–60. doi: 10.1016/j.clnu.2018.06.982 30025745

[95] Lai Y, Huang J, Yang M, Su J, Liu J, Che G. Seven-day intensive preoperative rehabilitation for elderly patients with lung cancer: a randomized controlled trial. J Surg Res. 2017;209:30–6. doi: 10.1016/j.jss.2016.09.033 28032568

[96] Lai Y, Su J, Qiu P, Wang M, Zhou K, Tang Y, et al. Systematic short-term pulmonary rehabilitation before lung cancer lobectomy: a randomized trial. Interact Cardiovasc Thorac Surg. 2017;25(3):476–83. doi: 10.1093/icvts/ivx141 28520962

[97] Licker M, Karenovics W, Diaper J, Frésard I, Triponez F, Ellenberger C, et al. Short-Term Preoperative High-Intensity Interval Training in Patients Awaiting Lung Cancer Surgery: A Randomized Controlled Trial. J Thorac Oncol. 2017;12(2):323–33. 27771425 10.1016/j.jtho.2016.09.125

[98] Lindbäck Y, Tropp H, Enthoven P, Abbott A, Öberg B. PREPARE: presurgery physiotherapy for patients with degenerative lumbar spine disorder: a randomized controlled trial. Spine J. 2018;18(8):1347–55. doi: 10.1016/j.spinee.2017.12.009 29253630

[99] Møller AM, Villebro N, Pedersen T, Tønnesen H. Effect of preoperative smoking intervention on postoperative complications: a randomised clinical trial. Lancet. 2002;359(9301):114–7. doi: 10.1016/S0140-6736(02)07369-5 11809253

[100] Nielsen PR, Jørgensen LD, Dahl B, Pedersen T, Tønnesen H. Prehabilitation and early rehabilitation after spinal surgery: randomized clinical trial. Clin Rehabil. 2010;24(2):137–48. doi: 10.1177/0269215509347432 20103575

[101] Rosenfeldt F, Braun L, Spitzer O, Bradley S, Shepherd J, Bailey M, et al. Physical conditioning and mental stress reduction—a randomised trial in patients undergoing cardiac surgery. BMC Complement Altern Med. 2011;11:20. doi: 10.1186/1472-6882-11-20 21385466 PMC3063826

[102] Sebio García R, Yáñez-Brage MI, Giménez Moolhuyzen E, Salorio Riobo M, Lista Paz A, Borro Mate JM. Preoperative exercise training prevents functional decline after lung resection surgery: a randomized, single-blind controlled trial. Clin Rehabil. 2017;31(8):1057–67. doi: 10.1177/0269215516684179 28730888

[103] Sørensen LT, Hemmingsen U, Jørgensen T. Strategies of smoking cessation intervention before hernia surgery—effect on perioperative smoking behavior. Hernia. 2007;11(4):327–33. doi: 10.1007/s10029-007-0229-0 17503161

[104] Sørensen LT, Jørgensen T. Short-term pre-operative smoking cessation intervention does not affect postoperative complications in colorectal surgery: a randomized clinical trial. Colorectal Dis. 2003;5(4):347–52. doi: 10.1046/j.1463-1318.2003.00450.x 12814414

[105] Steinmetz C, Bjarnason-Wehrens B, Baumgarten H, Walther T, Mengden T, Walther C. Prehabilitation in patients awaiting elective coronary artery bypass graft surgery—effects on functional capacity and quality of life: a randomized controlled trial. Clin Rehabil. 2020;34(10):1256–67. doi: 10.1177/0269215520933950 32546065 PMC7477776

[106] Villebro NM, Pedersen T, Møller AM, Tønnesen H. Long-term effects of a preoperative smoking cessation programme. Clin Respir J. 2008;2(3):175–82. doi: 10.1111/j.1752-699X.2008.00058.x 20298326

[107] Webb AR, Coward L, Meanger D, Leong S, White SL, Borland R. Offering mailed nicotine replacement therapy and Quitline support before elective surgery: a randomised controlled trial. Med J Aust. 2022;216(7):357–63. doi: 10.5694/mja2.51453 35267206 PMC9314866

[108] Hoogeboom TJ, Dronkers JJ, van den Ende CH, Oosting E, van Meeteren NL. Preoperative therapeutic exercise in frail elderly scheduled for total hip replacement: a randomized pilot trial. Clin Rehabil. 2010;24(10):901–10. doi: 10.1177/0269215510371427 20554640

[109] Karlsson E, Farahnak P, Franzén E, Nygren-Bonnier M, Dronkers J, van Meeteren N, et al. Feasibility of preoperative supervised home-based exercise in older adults undergoing colorectal cancer surgery—A randomized controlled design. PLoS One. 2019;14(7):e0219158. doi: 10.1371/journal.pone.0219158 31265476 PMC6605852

[110] Steffens D, Young J, Beckenkamp PR, Ratcliffe J, Rubie F, Ansari N, et al. Feasibility and acceptability of a preoperative exercise program for patients undergoing major cancer surgery: results from a pilot randomized controlled trial. Pilot and Feasibility Studies. 2021;7(1):27. doi: 10.1186/s40814-021-00765-8 33441181 PMC7805142

[111] van der Velde M, Valkenet K, Geleijn E, Kruisselbrink M, Marsman M, Janssen LM, et al. Usability and Preliminary Effectiveness of a Preoperative mHealth App for People Undergoing Major Surgery: Pilot Randomized Controlled Trial. JMIR Mhealth Uhealth. 2021;9(1):e23402. doi: 10.2196/23402 33410758 PMC7819776

[112] Molenaar CJL, Minnella EM, Coca-Martinez M, ten Cate DWG, Regis M, Awasthi R, et al. Effect of Multimodal Prehabilitation on Reducing Postoperative Complications and Enhancing Functional Capacity Following Colorectal Cancer Surgery: The PREHAB Randomized Clinical Trial. JAMA Surgery. 2023. doi: 10.1001/jamasurg.2023.0198 36988937 PMC10061316

[113] Almeida GJ, Khoja SS, Zelle BA. Effect of prehabilitation in older adults undergoing total joint replacement: An Overview of Systematic Reviews. Curr Geriatr Rep. 2020;9(4):280–7. doi: 10.1007/s13670-020-00342-6 33344110 PMC7745772

[114] Falz R, Bischoff C, Thieme R, Lässing J, Mehdorn M, Stelzner S, et al. Effects and duration of exercise-based prehabilitation in surgical therapy of colon and rectal cancer: a systematic review and meta-analysis. Journal of Cancer Research and Clinical Oncology. 2022. doi: 10.1007/s00432-022-04088-w 35695931 PMC9349170

[115] Bohannon RW, Crouch R. Minimal clinically important difference for change in 6-minute walk test distance of adults with pathology: a systematic review. J Eval Clin Pract. 2017;23(2):377–81. doi: 10.1111/jep.12629 27592691

[116] Wong J, Lam DP, Abrishami A, Chan MTV, Chung F. Short-term preoperative smoking cessation and postoperative complications: a systematic review and meta-analysis. Canadian Journal of Anesthesia/Journal canadien d’anesthésie. 2012;59(3):268–79. doi: 10.1007/s12630-011-9652-x 22187226

[117] Rigotti NA, Clair C, Munafò MR, Stead LF. Interventions for smoking cessation in hospitalised patients. Cochrane Database of Systematic Reviews. 2012(5). doi: 10.1002/14651858.CD001837.pub3 22592676 PMC4498489

[118] NHS. The NHS Long Term Plan. 2019.

[119] Tsimopoulou I, Pasquali S, Howard R, Desai A, Gourevitch D, Tolosa I, et al. Psychological Prehabilitation Before Cancer Surgery: A Systematic Review. Ann Surg Oncol. 2015;22(13):4117–23. doi: 10.1245/s10434-015-4550-z 25869228

[120] Roman M, Monaghan A, Serraino GF, Miller D, Pathak S, Lai F, et al. Meta-analysis of the influence of lifestyle changes for preoperative weight loss on surgical outcomes. British Journal of Surgery. 2019;106(3):181–9. doi: 10.1002/bjs.11001 30328098

[121] Carnethon MR, Khan SS. An Apparent Obesity Paradox in Cardiac Surgery. Circulation. 2017;135(9):864–6. doi: 10.1161/CIRCULATIONAHA.117.026856 28242638 PMC5331860

[122] Billings FTt, Pretorius M, Schildcrout JS, Mercaldo ND, Byrne JG, Ikizler TA, et al. Obesity and oxidative stress predict AKI after cardiac surgery. J Am Soc Nephrol. 2012;23(7):1221–8. doi: 10.1681/ASN.2011090940 22626819 PMC3380645

[123] Hafner S, Hillenbrand A, Knippschild U, Radermacher P. The obesity paradox and acute kidney injury: beneficial effects of hyper-inflammation? Critical Care. 2013;17(6):1023. doi: 10.1186/cc13152 24326122 PMC4059416

[124] Hoffmann TC, Glasziou PP, Boutron I, Milne R, Perera R, Moher D, et al. Better reporting of interventions: template for intervention description and replication (TIDieR) checklist and guide. BMJ: British Medical Journal. 2014;348:g1687. doi: 10.1136/bmj.g1687 24609605

[125] Grimmett C, Bradbury K, Dalton SO, Fecher-Jones I, Hoedjes M, Varkonyi-Sepp J, et al. The Role of Behavioral Science in Personalized Multimodal Prehabilitation in Cancer. Frontiers in Psychology. 2021;12. doi: 10.3389/fpsyg.2021.634223 33664701 PMC7921482

[126] National Institute for Health Care Excellence. Behaviour change: individual approaches: National Institute for Health and Care Excellence (NICE); 2014.

[127] Bennett KM, Scarborough JE, Pappas TN, Kepler TB. Patient Socioeconomic Status Is an Independent Predictor of Operative Mortality. Annals of Surgery. 2010;252(3). doi: 10.1097/SLA.0b013e3181f2ac64 20739856

[128] Jerath A, Austin PC, Ko DT, Wijeysundera HC, Fremes S, McCormack D, et al. Socioeconomic Status and Days Alive and Out of Hospital after Major Elective Noncardiac Surgery: A Population-based Cohort Study. Anesthesiology. 2020;132(4):713–22. doi: 10.1097/ALN.0000000000003123 31972656

[129] Saeed SA, Masters RM. Disparities in Health Care and the Digital Divide. Current Psychiatry Reports. 2021;23(9):61. doi: 10.1007/s11920-021-01274-4 34297202 PMC8300069

[130] NHS England. 2019/20 National Cost Collection Data Publication 2020 [Available from: https://www.england.nhs.uk/publication/2019-20-national-cost-collection-data-publication/.

[131] NHS South Central and West (SCW) Commissioning Support Unit. Prehab for Cancer Evaluation 2022.

