# Supplementary material for: The effect of preoperative behaviour change interventions on pre- and post-surgery health behaviours, health outcomes, and health inequalities in adults: A systematic review and meta-analyses
Source: PLoS One. 2023 Jul 5;18(7):e0286757. doi: 10.1371/journal.pone.0286757 (PMC10321619; doi:10.1371/journal.pone.0286757)
Supplement: S1 File — (DOCX) [file pone.0286757.s001.docx]

**Supplementary file 1. PRISMA checklist**

| **Section and Topic** | **Item #** | **Checklist item** | **Location where item is reported** |
| --- | --- | --- | --- |
| **TITLE** | | |  |
| Title | 1 | Identify the report as a systematic review. | Pg 1 |
| **ABSTRACT** | | |  |
| Abstract | 2 | See the PRISMA 2020 for Abstracts checklist. | Pg 2 |
| **INTRODUCTION** | | |  |
| Rationale | 3 | Describe the rationale for the review in the context of existing knowledge. | Pg 4-5 |
| Objectives | 4 | Provide an explicit statement of the objective(s) or question(s) the review addresses. | Pg 5 |
| **METHODS** | | |  |
| Eligibility criteria | 5 | Specify the inclusion and exclusion criteria for the review and how studies were grouped for the syntheses. | Pg 5-8 |
| Information sources | 6 | Specify all databases, registers, websites, organisations, reference lists and other sources searched or consulted to identify studies. Specify the date when each source was last searched or consulted. | Pg 7 |
| Search strategy | 7 | Present the full search strategies for all databases, registers and websites, including any filters and limits used. | Supp file 2 |
| Selection process | 8 | Specify the methods used to decide whether a study met the inclusion criteria of the review, including how many reviewers screened each record and each report retrieved, whether they worked independently, and if applicable, details of automation tools used in the process. | Pg 7 |
| Data collection process | 9 | Specify the methods used to collect data from reports, including how many reviewers collected data from each report, whether they worked independently, any processes for obtaining or confirming data from study investigators, and if applicable, details of automation tools used in the process. | Pg 7 |
| Data items | 10a | List and define all outcomes for which data were sought. Specify whether all results that were compatible with each outcome domain in each study were sought (e.g. for all measures, time points, analyses), and if not, the methods used to decide which results to collect. | Pg 7-8 |
|  | 10b | List and define all other variables for which data were sought (e.g. participant and intervention characteristics, funding sources). Describe any assumptions made about any missing or unclear information. | Pg 7-8 |
| Study risk of bias assessment | 11 | Specify the methods used to assess risk of bias in the included studies, including details of the tool(s) used, how many reviewers assessed each study and whether they worked independently, and if applicable, details of automation tools used in the process. | Pg 9 |
| Effect measures | 12 | Specify for each outcome the effect measure(s) (e.g. risk ratio, mean difference) used in the synthesis or presentation of results. | Pg 8 |
| Synthesis methods | 13a | Describe the processes used to decide which studies were eligible for each synthesis (e.g. tabulating the study intervention characteristics and comparing against the planned groups for each synthesis (item #5)). | Pg 7 |
|  | 13b | Describe any methods required to prepare the data for presentation or synthesis, such as handling of missing summary statistics, or data conversions. | Pg 7-8 |
|  | 13c | Describe any methods used to tabulate or visually display results of individual studies and syntheses. | Pg 7-8 |
|  | 13d | Describe any methods used to synthesize results and provide a rationale for the choice(s). If meta-analysis was performed, describe the model(s), method(s) to identify the presence and extent of statistical heterogeneity, and software package(s) used. | Pg 7-8 |
|  | 13e | Describe any methods used to explore possible causes of heterogeneity among study results (e.g. subgroup analysis, meta-regression). | Pg 8 |
|  | 13f | Describe any sensitivity analyses conducted to assess robustness of the synthesized results. | Pg 11-12 |
| Reporting bias assessment | 14 | Describe any methods used to assess risk of bias due to missing results in a synthesis (arising from reporting biases). | N/a |
| Certainty assessment | 15 | Describe any methods used to assess certainty (or confidence) in the body of evidence for an outcome. | N/a |
| **RESULTS** | | |  |
| Study selection | 16a | Describe the results of the search and selection process, from the number of records identified in the search to the number of studies included in the review, ideally using a flow diagram. | Pg 9-10 |
|  | 16b | Cite studies that might appear to meet the inclusion criteria, but which were excluded, and explain why they were excluded. | N/a |
| Study characteristics | 17 | Cite each included study and present its characteristics. | Supp Table 2 |
| Risk of bias in studies | 18 | Present assessments of risk of bias for each included study. | Pg 14-20 |
| Results of individual studies | 19 | For all outcomes, present, for each study: (a) summary statistics for each group (where appropriate) and (b) an effect estimate and its precision (e.g. confidence/credible interval), ideally using structured tables or plots. | Pg 13-20 |
| Results of syntheses | 20a | For each synthesis, briefly summarise the characteristics and risk of bias among contributing studies. | Pg 13-23 |
|  | 20b | Present results of all statistical syntheses conducted. If meta-analysis was done, present for each the summary estimate and its precision (e.g. confidence/credible interval) and measures of statistical heterogeneity. If comparing groups, describe the direction of the effect. | Pg 11-17 |
|  | 20c | Present results of all investigations of possible causes of heterogeneity among study results. | Pg 11-17 |
|  | 20d | Present results of all sensitivity analyses conducted to assess the robustness of the synthesized results. | Pg 11-12 |
| Reporting biases | 21 | Present assessments of risk of bias due to missing results (arising from reporting biases) for each synthesis assessed. | Pg 14-20 |
| Certainty of evidence | 22 | Present assessments of certainty (or confidence) in the body of evidence for each outcome assessed. | N/a |
| **DISCUSSION** | | |  |
| Discussion | 23a | Provide a general interpretation of the results in the context of other evidence. | Pg 20-22 |
|  | 23b | Discuss any limitations of the evidence included in the review. | Pg 22 |
|  | 23c | Discuss any limitations of the review processes used. | Pg 21-22 |
|  | 23d | Discuss implications of the results for practice, policy, and future research. | Pg 23 |
| **OTHER INFORMATION** | | |  |
| Registration and protocol | 24a | Provide registration information for the review, including register name and registration number, or state that the review was not registered. | Pg 5 |
|  | 24b | Indicate where the review protocol can be accessed, or state that a protocol was not prepared. | Pg 5 |
|  | 24c | Describe and explain any amendments to information provided at registration or in the protocol. | Supp Table 1 |
| Support | 25 | Describe sources of financial or non-financial support for the review, and the role of the funders or sponsors in the review. | Online submission |
| Competing interests | 26 | Declare any competing interests of review authors. | Online submission |
| Availability of data, code and other materials | 27 | Report which of the following are publicly available and where they can be found: template data collection forms; data extracted from included studies; data used for all analyses; analytic code; any other materials used in the review. | Online submission |

**Supplementary Table 1: Amendments to protocol**

| **Original** | **Amendment** | **Rationale** |
| --- | --- | --- |
| Interventions initiated at any time during the perioperative period | Only interventions initiated in the period before surgery | The original criteria yielded prehabilitation studies and these did not answer the research question |
| N/a | Excluded nutrition interventions that consisted of supplementation only | Dietary supplementation alone does not aim to promote dietary behaviour change and, therefore, these interventions do not address the research question |
| Inclusion of non-randomised controlled trials | Exclusion of non-randomised controlled trials | Given the vastness of the literature, additional criteria was applied to seek higher quality study designs and more robust evidence |
| Extraction of anthropometric or dietary outcomes for all studies in which they were reported | Exclude extraction of anthropometric or dietary outcomes where the intervention aimed to promote weight maintenance/gain (not weight loss) | As the review sought to address health behaviours of public health importance, the decision was made to only extract outcomes for studies promoting weight loss |
| Determine the certainty of evidence using the GRADE criteria | No completion of GRADE | Upon reflection, we deemed that it was not appropriate to apply GRADE to our broad research question; GRADE is not appropriate to exploratory research questions and its application could result in misinterpretation of the evidence |

**Supplementary file 2: OVID Medline search strategy**

1 health behavior/ or health risk behaviors/ or smoking cessation/ or smoking reduction/ or "tobacco use cessation"/ 82221

2 Behavior Therapy/ 28572

3 (behavio* adj2 chang*).mp. 53556

4 (lifestyle adj2 intervention*).mp. 8768

5 (behavio* adj2 modifi*).mp. 7405

6 (lifestyle adj2 chang*).mp. 11636

7 "health related behavior*".mp. 2605

8 "health related behaviour*".mp. 1457

9 (lifestyle adj2 modifi*).mp. 8612

10 "behavior change technique".mp. 78

11 diet.mp.ti,ab,kw. 175159

12 Diet.mp. 490426

13 Feeding Behavior/ 85578

14 eating behavio?r.mp. 7545

15 alcohol.ti,ab,kw. 266613

16 alcohol drinking/ or binge drinking/ 70314

17 smoking.ti,ab,kw. 234313

18 smoking/ or smoking reduction/ or tobacco smoking/ 143840

19 "tobacco use"/ or tobacco smoking/ 3621

20 exercise.mp. 371977

21 "physical activit*".mp. 124150

22 Preoperative Exercise/ 55

23 Exercise Therapy/ 42814

24 exercise/ or physical conditioning, human/ 119754

25 Prehabilitation.mp. 806

26 Preoperative rehabilitation*.mp. 109

27 Pre-operative rehabilitation*.mp. 25

28 1 or 2 or 3 or 4 or 5 or 6 or 7 or 8 or 9 or 10 or 11 or 12 or 13 or 14 or 15 or 16 or 17 or 18 or 19 or 20 or 21 or 22 or 23 or 24 or 25 or 26 or 27 1632153

29 Elective surgical procedure*.mp. 16049

30 General Surgery/ 39539

31 postoperative complications/ or pain, postoperative/ 413408

32 Operation.mp. 355198

33 Resection.mp. 304432

34 Surgical intervention*.mp. 69297

35 Surgical procedure*.mp. 439125

36 Surgical treatment*.mp. 159136

37 Excission.mp. 19

38 Abscission.mp. 1863

39 specialties, surgical/ or general surgery/ 43208

40 surgical procedures, operative/ or perioperative care/ 70071

41 Reconstructive Surgical Procedures/ 52737

42 Endoscopy/ 53956

43 Surgery.mp. 2748461

44 Reoperation/ 89490

45 Elective Surgical Procedures/ 15223

46 29 or 30 or 31 or 32 or 33 or 34 or 35 or 36 or 37 or 38 or 39 or 40 or 41 or 42 or 43 or 44 or 45 3218939

47 28 and 46 88126

48 limit 47 to (humans and ("adult (19 to 44 years)" or "young adult and adult (19-24 and 19-44)" or "middle age (45 to 64 years)" or "middle aged (45 plus years)" or "all aged (65 and over)" or "aged (80 and over)")) 47259

49 clinical trial/ or controlled clinical trial/ 551751

50 Randomized Controlled Trial/ or trial.mp. 1250525

51 49 or 50 1250525

52 48 and 51 6624

**Supplementary Table 2:** Characteristics of studies included in the review.

| **Study year, country** | **Design** | **n of participants** | **Surgical procedure** | **Main inclusion criteria** | **Main exclusion criteria** | **Mean age (years) (SD)** | **Percentage female** | **Mean BMI (SD)** | **Socioeconomic status** | **Ethnicity** |
| --- | --- | --- | --- | --- | --- | --- | --- | --- | --- | --- |
| Abdelaal et al. 2017, Egypt (38) | RCT | 50 | Upper abdominal surgery | BMI >30; No past surgical intervention in upper abdomen; Good general condition | Unstable comorbidities | Median (IQR):  Intervention: 55.5 (49–67)  Control: 52 (47–65) | 56.0% | I: BMI 30-40: 77%  BMI > 40: 23% C: BMI 30-40: 87% BMI > 40: 13% | NR | NR |
| Arthur et al. 2000 (46), Canada | RCT | 249 | CABG-surgery | First CABG surgery;  at least 10 weeks away from surgery; ‘low risk’ patients | Combined CABG and valve surgery; unable to participate in exercise due to geographical or physical limitations | 62.8 (8.1) | 14.6% | NR | Mean 13.3 years of education; 20.3% employed | NR |
| Barakat et al. 2016 (64), UK | RCT | 136 | Open or endovascular AAA repair surgery | > 18 years old; AAA ≥ 5.5 cm | Presence of factors that may limit exercise e.g., severe musculoskeletal disorders; patients with thoracic aortic aneurysms | 73.4 (7.2) | 10.5% | 27.0 (3.9) | NR | NR |
| Barberan-Garcia et al. 2018 (91) and Barberan Garcia et al. 2019 (90), Spain | RCT | 144 | Major abdominal surgery | High risk of surgical complications, schedule allowing at least 4 weeks for prehab intervention | Nonelective surgery, unstable cardiac / respiratory disease, locomotor or cognitive limitations precluding participation in the intervention | 71 (10.5) | 24.8% | 21.5 (7) | NR | NR |
| Berkel et al., 2021 (92), Netherlands | RCT | 74 | Colorectal Resection | ≥ 60 years old; ventilatory anaerobic threshold <11 mL/kg/min at baseline; score ≤7 Metabolic equivalents on the veteran specific activity questionnaire (VSAQ) | NR | 73.5 (6.5) | 47.0% | 30.2 (4.5) | NR | NR |
| Benzo et al. 2012 (75), USA | RCT | Stud 1: 9  Study 2: 19 | Lung resection | Moderate to severe COPD | NR | NR | Study 2: 52.6% | NR | NR | NR |
| Bhatia et al. (93), 2019, Switzerland | RCT | 151 | Primary lung resection | Suspected or proven NSCLC stage IIIA or less | Contraindications to perform CPET; limitations to adhering to the rehabilitation programme | 64 (11.5) | 39.7% | 24.7 (4.3) | NR | NR |
| Bousquet-Dion et al (47). 2018, Canada | RCT | 80 | Colorectal Resection | Adults; Scheduled for colon or rectal cancer resection | Metastatic cancer Did not speak English or French  Conditions that contraindicated exercise | Median (IQR): I: 74 (67.5-78.0) C: 71 (54.5-74.5) | 27.0% | 28.0 (4.3) | NR | NR |
| Carli et al., 2020 (48), Canada | RCT | 120 | Colorectal Resection | ≥ 65 years; frailty | Metastatic cancer Conditions that contraindicated exercise | Median (IQR):  I:78 (72- 82) C: 82 (75- 84) | 53.0% | Median (IQR): I: 24.9 (23.0 - 30.1) C: 26.4 (23.8 - 30.6) | NR | NR |
| Demark-Wahnefried et al (78). 2017, USA | RCT | 40 | Radical prostatectomy | Men;  >19 years; BMI 25–50; biopsy-confirmed prostate cancer; scheduled for radical prostatectomy > 3 weeks | Other active malignancies or health conditions that affected body weight or precluded unsupervised PA; Taking part in weight loss programme | 60.1 (6.3) | 0.0% | 31.4 (4.5) | High school graduate (20%) Some college/technical n= 30% College graduate 20% Post graduate 30% | African/American: 30% Non-Hispanic White 70% |
| D'Lima et al.(77), 1996, USA | RCT | 30 | Knee replacement surgery | > 55 years; Primary diagnosis of arthritis; Residence within convenient distance of the facility | Cognitive, psychological, or language impairment | 69.9 (5.9) | 46.7% | Weight (kg): 82.5 (14.8) | NR | NR |
| Dunne et al.(65), 2016, UK | RCT | 38 | Liver resection | ≥18 years; resectable colorectal liver metastasis; could complete the exercise programme before the proposed surgery date | Patients with pre-existing chronic liver disease | Median (IQR): 62 (54–69) | 29.7% | 29⋅5 (4⋅1) | NR | NR |
| Ferreira et al., 2020 (49), Canada | RCT | 124 | Lung resection | >18 years; scheduled for non-small cell lung cancer resection | Metastatic cancer; did not speak English or French; conditions that contraindicated exercise | 70.0 (9.4) | 46.3% | Median (IQR): I: 27.9 (25.9-32.8) C: 26.9 ( 22.9-30.2) | NR | NR |
| Ferreira et al. 2021(50) and Lawson et al. 2021 (51), Canada | Pilot feasibility RCT | 34 | Video-assisted thoracic surgery or open thoracotomy surgery | Consecutive adult patients scheduled for elective video-assisted thoracic surgery or open thoracotomy surgery of lung cancer stages I, II or IIIa | Recent chemotherapy, comorbidities contraindicating exercise, walking aids other than a cane, glomerular filtration rate <30 mL/min/ m , allergy to milk or seafoods | Median (IQR):  I: 67 (63.3 - 72)  C: 69 (66.8 - 73.3) | 47.1% | Median (IQR):  I: 26.6 (23–32.6)  C: 28.3 (23.6–31.2] ) | NR | NR |
| Fulop et al., 2021(36), Hungary | RCT | 184 | Colorectal resection | ≥ 18 years; required colorectal resection surgery | Emergency surgical procedures; palliative resection; ostomy or internal bypass due to peritoneal carcinomatosis; extensive metastatic or unresectable disease. | Median (IQR):  I: 70 (60–75) C:70 (64–75) | 49.0% | 27.9 (5.5) | NR | NR |
| Furze et al, 2009 (66), UK | RCT | 204 | CABG surgery | All patients admitted to the routine waiting list for CABG at a cardiothoracic centre | Exercise induced arrhythmias; loss of systolic BP greater than 20 mm Hg during exercise stress testing; unstable angina psychiatric problems, dementia, life threatening comorbidities | 64.8 (8.7) | 19.6% | 29.0 (4.6) | NR | NR |
| Gillis et al., 2014 (94), Chen et al., 2016 (52), Canada | RCT | 89 | Colorectal resection | Scheduled for curative resection of nonmetastatic colorectal cancer | Premorbid conditions that contraindicated exercise | 65.9 (11.3) | 37.7% | 27.7 (4.4) | NR | NR |
| Goodman et al., 2008 (67), UK | RCT | 188 | CABG surgery | Require cardiac bypass surgery; Risk factors: BP 140 mmHg systolic or 80 mmHg diastolic, non-fasting serum cholesterol 4 mmol/L or BMI >28 | Lived outside the designated geographical area; life-threatening significant non-cardiovascular disease | 64.8 (SD NR) | 18.6% | Body Mass Index ≥ 28: 61.7% | NR | NR |
| Goodney et al. 2017 (79), USA | Pilot feasibility cluster RCT | 156 | Vascular surgery | >18 years; smoker; peripheral arterial disease | NR | 61.0 (7.7) | 28.0% | NR | Education:  I: < High school = 9.2% C: < High school = 15.4% | NR |
| Haddock et al. 1997 (68), UK | Pilot/Feasibility RCT | 60 | NR, but patients recruited through day surgery and gynaecology | Smokers | Non-smokers | n = 40 (66%) aged ≥40 years | 68.3% | NR | NR | NR |
| Hoogeboom et al 2010 (108), Netherlands | Pilot/Feasibility RCT | 21 | Total hip replacement | >70 years; scheduled for elective primary total hip replacement due to end-stage osteoarthritis; waiting time > 3 weeks; 2 on the Clinical Frailty Scale | Unable to communicate in Dutch; severe heart disease | 76 (4.1) | 66.6% | 26.7 (3.4) | NR | NR |
| Karlsson et al, 2019 (109), Sweden | Pilot/Feasibility RCT | 23 | Colorectal surgery | >70 years; understand and speak Swedish; scheduled for surgery due to colorectal cancer | A health status that prohibits physical exercise, if prolonged preoperative period was consistent with a medical risk; if the participant lived outside the catchment area of the included primary care units | Median (IQR): 76.0 (73–84) | 62.0% | NR | NR | NR |
| Kim et al. 2009 (53), Canada | Pilot/Feasibility RCT | 21 | Colorectal surgery | Colorectal  resection for benign, non-disseminated colon and rectum cancers, colon reconstruction, fibrostenotic | American Society of Anesthesiologists health status  class 4-5 with other co-morbid medical conditions preventing study participation; high risk for a cardiac complication during exercise; sepsis; chemotherapy  or radiotherapy during six months prior to the date of surgery | 53.3 (13) | 38.1% | 26.2 (4.8) | NR | NR |
| Kim et al. 2021 (80), USA | Pilot RCT | 43 | Total knee replacement | ≥ 50 years, ≤ 50th percentile on mobility assessment tool-short form | Undergoing TKA for indication other than osteoarthritis, revision or bilateral surgery, afraid of water or not willing to undergo water exercise, currently exercising > 3 x/week | 67.2 (6.1) | 44.0% | 32.4 (6.3) | NR | 74% White  21% Black |
| Lai et al., 2017a (96), China | RCT | 101 | Lung lobectomy | age >75 years; BMI >30; >20 pack-year smoking history; | Contraindications to the physical rehabilitation; Unstable angina pectoris; Aneurysm; Recent history (<90 days) of haemoptysis; Severe arrhythmia; Musculoskeletal disorders; Mental disorders; Not undergoing surgery; Undergoing sublobar resection or pneumonectomy; Diagnosis other than NSCLC | 64.2 (7.4) | 45.0% | 25.7 % >30 BMI | NR | NR |
| Lai et al., 2017b (95), China | RCT | 60 | Lung lobectomy | Diagnosis of primary non small-cell Lung cancer; No surgical contraindication | Myocardial infarction; Cerebrovascular accident (<1 y),  Unstable angina pectoris;  Aneurysm; Hemoptysis (<90 d); Severe arrhythmia;  Musculoskeletal; Mental disorders | 73.4 (5.3) | 43.0% | 16.6% >30 BMI | NR | NR |
| Lee et al., 2013 (54), Lee at al. 2015 (55), Canada | RCT | 168 | General, gynecologic, urologic, ophthalmologic, otolaryngologic, and orthopedic surgery | >18 years; current daily smoker;  presented to the preadmission clinic > 3 weeks preoperatively | Pregnant or breastfeeding; active participation in another smoking cessation trial;  inability to consent due to severe mental illness or dementia | 47.5 (12.8) | 54.8% | 27.5 (5.4) | NR | NR |
| Liang et al 2018 (81), Bernadi et al. (76), 2020, USA | RCT | 118 | Ventral hernia repair | BMI 30-40; desired repair;   ventral hernia measuring between 3-20 cm in width | Severe comorbid conditions limiting survival to less than two years;  indications for urgent/emergent surgery, enterocutaneous fistula, intermittent intestinal incarceration, or local or systemic infection;  pregnancy | 49.5 (10.1) | 70.3% | 36.8 (2.6) | NR | NR |
| Licker et al., 2017 (97), Switzerland | RCT | 151 | Lung resection | NSCLC, stage IIIA or less. | Any contraindication to performing CPET | 64 (11.5) | 39.7% | 24.7 (4.3) | NR | NR |
| Ligibel et al. (82), 2016, USA | RCT | 48 | Breast surgery | Diagnosis of invasive breast cancer; planning to undergo primary breast surgery; participation of fewer than 90 minutes of MVPA per week | Taking hormonal agents, diabetes or distant breast cancer metastases; planning to undergo neoadjuvant systemic therapy; uncontrolled cardiac disease or other contraindications to moderate-intensity exercise | 52.6 (8.8) | 100.0% | 30 (6.7) | NR | Hispanic: 5 (11%)  Non-hispanic: 38 (78%) Unknown: 5 (11%) |
| Liljensoe et al. (85), 2019, Denmark | RCT | 76 | Total knee replacement surgery | BMI >30; scheduled for primary TKR; motivated for weight loss | Rheumatoid arthritis; planned bariatric surgery | Mean (range): I: 65 (46-81) C: 65 (46-85) | 71.1% | Mean (95%CI) I: 31.6 (30.6, 32.6)  Cl: 31.2 (29.8, 32.6) | Unskilled worker: 41% Skilled worker: 48% Bachelor's/master's degree: 11% | NR |
| Lindbäck et al., 2018 (98), Sweden | RCT | 197 | Lumbar spine surgery | 25-80 years; presence of LBP and/or leg pain due to disc herniation, spinal stenosis, spondylolisthesis; DDD; diagnosis. | In need of acute surgery or re-surgery on the same level;  had severe spinal pathology or other severe diagnoses | 59.0 (12.5) | 53.0% | NR | NR | NR |
| Lindstrom et al., 2008 (42), Sweden | RCT | 102 | General and orthopaedic surgery | 18 - 79 years; daily smoker | Alcohol or drug abuse; pregnancy; severe mental illness; dementia | Median (IQR):  I: 55.0 (46-60) C: 57.5 (49-64) | 47.1% | Median (IQR):  I: 26.0 (24-30) C: 25.0 (23-29) | NR | NR |
| Liu et al. (37), 2020, China | RCT | 73 | Thoracoscopic lobectomy | <70 years; newly suspected or confirmed NSCLC, clinical stage I–III; scheduled for VATS lobectomy at PUMCH | American Society of Anesthesiologists (ASA) grade >III; received neoadjuvant therapy; premorbid conditions that contraindicated any items in the program | 56.2 (9.5) | 68.5% | 23.6 (3.1) | Education level above high school: 48 (65.8%) | NR |
| Lotzke et al. (86), 2019, Sweden | RCT | 118 | Lumbar fusion surgery | 18- 70 years; scheduled for lumbar fusion surgery. | Previous decompression surgery for spinal stenosis, spinal malignancy, dominating radiculopathy, confirmed neurological or rheumatic disorder;  deformities in the thoracolumbar spine | 45.7 (8.3) | 53.4% | 26.3 (3.7) | Level of education: Elementary school: 5.9% High school: 43.2% University: 35.6% Vocational education: 14.4% | NR |
| McHugh et al., 2001(69), UK | RCT | 98 | CABG surgery | Patients on the elective CABG waiting list | NS | Median (IQR): I: 61.1 (35–77)  C: 63.0 (42–76) | 24.5% | 28.0 (3.5) | NR | NR |
| McIsaac et al. 2022 (56), Canada | RCT | 204 | Abdominal or urological surgery | ≥60 years, score of ≥ 4/9 on Frailty Scale | Expected date of surgery < 21 days from day of enrolment | 74 (5.7) | 56.60% | NR | NR | NR |
| Minnella et al., 2018 (57), Canada | RCT | 68 | Esophagogastric resection | >18 years Referred electively for management of nonmetastatic esophagogastric cancer | Severe mental health issues;  co-morbidities | 67.6 (9.5) | 35.5% | 25.9 (4.8) | NR | NR |
| Minnella et al., 2019 (58), Canada | Pilot/Feasibility RCT | 70 | Radical cystectomy | > 18 years,  Scheduled for elective radical cystectomy for nonmetastatic bladder cancer; No medical conditions that precluded safe training. | < 4 weeks until surgery | 67.9 (10.2) | 30.0% | 27.4 (4.7) | NR | NR |
| Molenaar et al., 2023 (112) , Netherlands | RCT | 269 | Colorectal surgery | Can wait 4 weeks for surgery | Metastases or another primary tumour | Median (IQR):  Intervention: 69 (60-77)  Control: 71 (60-76) | 45.0 % | Median (IQR):  Intervention: 26.3 (24.2 – 30.2)  Control: 27.6 (24.7 – 31.7) | NR | NR |
| Moller et al., 2002 (99), Villebro, 2008 Denmark (106) | RCT | 120 | Hip or knee alloplasty | Daily smoker | Weekly alcohol intake > 35 units | Median (IQR): I: 66 (41-83) C: 64 (30-85) | 57.4% | Median (IQR): I: 27 (15-43) C: 26 (17-44) | NR | NR |
| Moug et al., 2019 (70), UK | Pilot/Feasibility RCT | 48 | Rectal surgery | No evidence of metastatic disease; recommended long-course NACRT followed by potentially curative surgery | Metastatic disease; unable to perform walking intervention; already achieving their recommended guidelines for physical activity/ week | 65.9 (10.5) | 35.0% | 18.8% had obesity (BMI ≥ 30) | 19% in most deprived quintile of Scottish IMD | 96% white |
| Nguyen et al. 2022 (87), France | RCT | 262 | Total knee replacement | Aged 50-85 years; knee osteoarthritis | TKA for indication other than osteoarthritis; history of homolateral TKR, history of chronic inflammatory joint disease, cognitive/behavioural disorder | 68.6 (8.0) | 68.0% | 29.4 (5.2) | Higher education level 97 (37%) | NR |
| Nielsen et al., 2010 (100), Denmark | RCT | 73 | Lumbar spinal surgery | > 18 years | Liver disease; nephropathy; pregnancy | Median (IQR): I: 48 (31-80) C: 52 (23-88) | 59.0% | Median (IQR): I: 25 (21-33) C: 26 (17-33) | NR | NR |
| Northgraves et al 2020 (71), UK | Pilot/Feasibility RCT | 22 | Colorectal surgery | Patients with benign disease;  preoperative period of 2 weeks was expected | Known cardiac or uncontrolled metabolic or respiratory condition precluding exercise; hypertension;   pre-existing severe physical disability preventing participation study components | 63.8 (11.4) | 47.6% | 29.0 (5.0) | NR | NR |
| Onerup et al., 2021 (88), Sweden | RCT | 761 | Colorectal surgery | ≥ 20 years | Emergency surgery; local surgery;  cytoreductive surgery with subsequent hyperthermic intraperitoneal chemotherapy (HIPEC) | 68 (11) | 40.0% | 26 (4.5) | 33% attended University | 13% born abroad |
| Pehlivan et al. 2011 (84), Turkey | RCT | 60 | Lung resection | No major cardiac morbidity | NR | 54.4 (8.5) | NR | 23.6 (3.2) | NR | NR |
| Ratner et al. 2004 (59), Canada | RCT | 237 | Surgery from any of the 10 surgical services | Current smokers;  expected to remain in the hospital for at least 24 hr following surgery | NR | 49.7 (13.6) | 51.0% | NR | Education and income: I: 29.1% attended University; 33.9% ≤ $29,000 C: 22.5% attended University; 27.7% ≤ $29,999 | NR |
| Rengel et al (83). 2021, USA | Pilot/Feasibility RCT | 32 | Major non-cardiac surgery | Requiring ≥3 days hospitalisation | <1 week remained before surgery; pre-existing conditions preventing participation in resistance-based physical exercise; no access to the internet | Median (IQR): I: 62 (50-69) C: 56 (44-66) | NR | NR | NR | NR |
| Rooks et al.(39), 2006, USA | RCT | 108 | Total hip and knee arthroplasty surgery | Scheduled unilateral primary THA or TKA for advanced osteoarthritis; understand and speak English;   8 –12 week time between enrolment and surgery | Patients with inflammatory arthritis, Parkinson’s disease, or any medical condition in which a moderate level of exercise was contraindicated; scheduled to have bilateral joint replacements | 64.1 (8.6) | 55.5% | 31.6 (7.4) | NR | NR |
| Rosenfeldt et al. 2011(101), Australia | RCT | 117 | CABG or valve surgery | Patients undergoing elective coronary artery bypass graft (CABG) and/or valve surgery at the Alfred Hospital | Had urgent or emergency surgery; Severe aortic valve stenosis;  NYHA class IV heart failure | Median (IQR):  I: 62.5 (59.0-68.5) C: 68 (58.0-77.0) | 25.6% | NR | NR | NR |
| Sadr Azodi et al. 2008(43), Sweden | RCT | 102 | Hip or knee arthroplasty, inguinal or umbilical primary hernia repair, laproscopic cholecystectomy surgery | 18-79 years; daily smoker; | Alcohol or drug abuse; pregnancy; severe mental illness or dementia | Median (IQR): I: 55 (46-60) C: 57.5 (49-64) | 47.1% | Median (IQR):  Intervention: 26 (24-30) Control: 25 (23-29) | University educated: 22.5% Married or had a partner: 32.4% | NR |
| Santa-Mina et al. 2018(60) and Au et al. 2019(61), Canada | Pilot/Feasibility RCT | 86 | Radical prostatectomy | Male; 40 - 80 years; Localized prostate cancer; Consented for RP;  Proficient in English or French; Able to exercise | Severe coronary artery disease; Congestive heart failure;  Uncontrolled pain; neurological or musculoskeletal co-morbidity inhibiting exercise; diagnosed psychotic, addictive, or major cognitive disorders | 61.7 (7.5) | 0.0% | 27.1 (4.3) | Annual income: < $40, 26.7%  Education:  <High school 8.1% | White 69.8% Black 12.8% Other/NR 17.5% |
| Sawatzky et al. 2014 (62), Canada | Pilot/Feasibility RCT | 17 | CABG surgery | Minimum estimated four week wait-time; No history of unstable angina, myocardial infarction in the last week, or dementia;  Sedentary prior to enrolment | Physical limitations or exercise-induced arrhythmias | 63.5 (8.1) | 20.0% | 30.8 (3.6) | NR | NR |
| Sebio Garcia et al. 2017(102), Spain | RCT | 40 | Lung resection | >18 years; suspected or confirmed diagnosis of NSCLC;  At least one of: (a) FEV1 ⩽80% of  predicted value; (b) BMI >30; (c age >75years or (d) two or more co-morbidities | Neoadjuvant therapy with chemo- or radiotherapy in the 6 months prior to surgery; inability to perform the exercise training | 70.1 (7.9) | 9.0% | 27.4 (3.5) | NR | NR |
| Snowden et al. 2020 (72), UK | Pilot/Feasibility RCT | 68 | Primary hip or knee arthroplasty surgery | >18 years; listed for elective primary hip or knee arthroplasty; risky drinking | Score <5 on AUDIT-C questionnaire; do not drink alcohol; psychiatric disorder; possible alcohol dependency; surgery too soon or sequential surgeries | 66.2 (SD NR) | 19.1% | 31.7 (SD NR) | NR | White British: 98.5% |
| Sorensen et al. 2003 (104), Denmark | RCT | 60 | Colorectal surgery | Daily smoker; scheduled for an open colonic or rectal operative procedure with formation of anastomosis | Inflammatory bowel disease | Median (IQR):  I: 65 (34-87) C: 66 (42-83) | 35.1% | 23.7 (5.1) | NR | NR |
| Sorensen et al. 2007 (103), Denmark | RCT | 213 | Incisional or inguinal day-case herniotomy surgery | Daily smoker; elective open incisional or inguinal day-case herniotomy | Cancelled surgery; did not answer questionnaires about perioperative smoking behaviour | Median (IQR):  Standard group: 54 (42-64) Reminder group: 54 (45-65) C: 56 (46-66) | 11.7% | 24.9 (4.0) | NR | NR |
| Steffens et al. 2021 (110), Australia | Pilot/Feasibility RCT | 22 | Elective pelvic exenteration, cytoreductive Surgery and hyperthermic intraperitoneal chemotherapy (HIPEC) | 18 - 80 years; underwent elective pelvic exenteration or CRS & HIPEC; presented to GI surgeon > 2 weeks before surgery | Cognitive impairment; co-morbidity preventing participation in exercise; current participation in an active exercise program | Median and IQR:  I: 66 (46-70)  C: 62 (48 -72) | 45.0% | NR | NR | NR |
| Steinmetz et al. 2020 (105), Germany | RCT | 230 | CABG surgery | Stable coronary artery disease, exercise-induced angina pectoris threshold ⩾50W | Unstable angina pectoris and/or myocardial infarction during the last two weeks; left main stem stenosis >50%; exercise induced angina threshold or ischemia at 60 minutes | 67.1 (8.4) | 10.3% | 28.7 (4.4) | NR | NR |
| Tew et al. 2017 (73), UK | Pilot/Feasibility RCT | 53 | AAA repair surgery | Under consideration for open or endovascular repair for AAA | AAA managed non-operatively; contraindication to exercise training; BMI < 20 or >40 | 74.7 (5.9) | 5.7% | 26.7 (3.8) | NR | NR |
| Thomsen et al. 2010 (44), Denmark | RCT | 130 | Breast surgery | Daily smoker; women | Alcohol intake exceeding 35 units/week; substance abuse; psychiatric disease; dementia; American Society of Anaesthesiologists’ (ASA) physical status classification-class >3; preoperative neoadjuvant chemotherapy | Median (IQR) Intervention: 57.5 (35-79) Control: 56.5 (36-82) | 100.0% | Median (IQR) Intervention: 24.2 (15.2-36.2) Control: 23.1 (16.5-37.9) | NR | NR |
| van der Velde et al. 2021 (111), Netherlands | Pilot/Feasibility RCT | 86 | Major elective surgery | Indicated for post op stay for at least 2 nights; one or more risk behaviours | No access to mobile device | Median (IQR):  61 (51-68) | 49.0% | Median (IQR):  25.8 (23.9-28.3) | NR | NR |
| Waller et al. 2021 (74), UK | Pilot/Feasibility RCT | 22 | Major abdominal surgery | Have at least 2 weeks to scheduled procedure | Health conditions that prevent safe participation in a home-based exercise programme; active users of an activity monitoring smartwatch; completed 150 mins of physical activity per week | Mean (95% CI): I: 55.5 (49.3, 61.7) C: 61.0 (53.1, 68.9) | 50.0% | Mean (95% CI): I: 30.0 (25.6, 34.4) C: 27.8 (23.4, 32.2) | NR | NR |
| Webb et al. 2022 (107), Australia | RCT | 516 | Most surgical specialties apart from cardiac and neurosurgery | Smokers | Allergic to NRT, known to be pregnant or breastfeeding, weighing less than 45 kg, undergoing endoscopy or surgery within ten days of listing | 49.8 (15.3) | 57.6% | NR | NR | NR |
| Wolfenden et al. 2005 (89), Australia | RCT | 210 | Non-cardiac elective surgery | Pre-existing health problems that required a pre-operative assessment; smoker | Pregnancy | 43.2 (13.9) | 61.9% | NR | High school education: Intervention: 60%  Control: 65% | NR |
| Wong et al 2017 (63), Canada | RCT | 296 | General surgery, orthopedics, urology, plastic surgery, vascular surgery, otolaryngology, ophthalmology, and neurosurgery | Scheduled for surgery within next 7-60 days; patients who smoked at least 10 cigarettes/ day during the previous year and had no period of smoking abstinence longer than 3 months in the past year | Pregnant or breastfeeding; major psychological disorder within the previous year; use of nicotine replacement or bupropion within the previous 3 months; CVD or a serious/unstable disease within the past 6 months; drug/alcohol abuse or dependence; use of tobacco products other than cigarettes or marijuana within the previous month; cognitive impairment | 51.8 (12.5) | 37.5% | 26.3 (6.4) | NR | NR |

I: Intervention, C: Comparator

AAA = abdominal aortic aneurysm; CABG = coronary artery bypass graft; PAD = peripheral arterial disease, NACRT = neoadjuvant chemoradiotherapy, TKR= Total knee replacement. THR = total hip replacement.

**Supplementary Table 3:** Intervention characteristics of included studies

| Study and Year | Multi- or single behaviour | Behaviours targeted | Setting | Intervention (Main behavioural components) | Delivered by | Duration | Timepoint in relation to surgery | Comparator group |
| --- | --- | --- | --- | --- | --- | --- | --- | --- |
| Abdelaal et al. 2017(38) | Single | PA | Home-based/ hospital (outpatients) | 10 mins walking 2x/day, 4x/week plus 2x physical therapy sessions/ week focusing on strengthening exercises | Physiotherapist | 2 weeks | Immediately before surgery | No physical therapy |
| Arthur et al. 2000 (46) | Single | PA | “Supervised environment” | Prescribed exercise training 2 x/week; education and reinforcement; and monthly  nurse-initiated telephone calls to answer questions and provide reassurance. | Nurses, kinesiologists, dietitians | Mean 8.3 weeks | NR | Usual care |
| Barakat et al. 2016 (64) | Single | PA | Hospital (outpatient) | 1 hour combined resistance and cardio exercise sessions 3x/week | NR | 6 weeks | Immediately before surgery | Instructed to continue with normal lifestyle and avoid any additional, unsupervised exercises |
| Barberan-Garcia et al. 2018 (91) and Barberan Garcia et al. 2019 (90) | Single | PA | Community setting, NR where supervised exercise was performed | Motivational interview & personalized programme, 40 mins supervised high-intensity endurance exercise training 1-3x/week. Given pedometer to increase steps/day & functional exercises. | Physiotherapist | 6 weeks | NR | Usual care |
| Benzo et al. 2012 (75) | Single | PA | NR | Study 1: exercise prescription according to American Thoracic Society/European Respiratory Society statement on pulmonary rehabilitation.  Study 2: aerobic endurance exercise and resistance 2x/day; daily walking | NR | Study 1: 4 weeks  Study 2: 1 week | NR | Usual care |
| Berkel et al., 2021 (92) | Single | PA | Physiotherapy clinic | Personalised exercise programme (cardio and strengthening exercises) 3x/week for 3 weeks & usual care | Physiotherapist | 3 weeks | NR | Usual care |
| Bhatia et al. (93), 2019 | Single | PA | NR | 30 mins supervised HIIT training on cycle ergometer 3x/week | Physiotherapist | 2-3 weeks | NR | Usual care |
| Bousquet-Dion et al (47). 2018 | Multi | PA, Diet | Home-based/ hospital (outpatients) | 30 mins moderate intensity aerobic activity 3/4 days/week and resistance training 3–4x/week & nutritional counselling. Given pedometer to encourage walking. | Kinesiologist, dietitian | Approx. 4 weeks | Immediately before surgery | Usual care |
| Carli et al., 2020 (48) | Multi | Smoking, PA, Diet, Alcohol | Home-based/ hospital (outpatients) | 30 mins supervised aerobic exercise & resistance exercise once/week; Personalised home-based program of aerobic activities and resistance training. Dietetic counselling on balanced diet and adequate protein intake; counselling on smoking and alcohol +/- NRT | Kinesiologist, nutritionist | 4 weeks | Immediately before surgery | Same as for intervention group but post-surgery for 4 weeks |
| Demark-Wahnefried et al (78). 2017 | Multi | PA, Diet | Hospital (outpatient) | Energy-restricted diet (deficit of 1000 calories per day), meeting with dietitians 2x/week, aerobic PA (up to 30 mins daily) and daily self-weighing. | Dieticians, exercise physiologists | Average 50 days | NR | Waitlist control - received intervention post-surgery after study completion |
| D'Lima et al.(77), 1996 | Single | PA | Physiotherapy clinic | 45 mins exercise sessions 3x/week for 18 sessions with group one focusing on resistance exercise and group 2 on aerobic exercise. | Physical therapist | 5 weeks | Approx. 1 week before surgery | Usual care, post-operative rehab programme. |
| Dunne et al.(65), 2016 | Single | PA | NR | 30 mins interval exercise aerobic sessions 12 times over 4 weeks | NR | 4 weeks | Immediately before surgery | Usual care |
| Ferreira et al., 2020 (49) | Multi | Smoking, PA, Diet | Home-based | 30 mins aerobic training 3 days/week. Resistance training 3 days/week & individualized nutritional care & smoking cessation | Kinesiologist, dietitian | 4 weeks | Within one week of surgery | Same as for intervention group but post-surgery for 8 weeks |
| Ferreira et al. 2021(50) and Lawson et al. 2021 (51) | Multi | Smoking, PA | Hospital-based | 1 session of 30 min aerobic training and 30 mins resistance training 1x/week; referred to meet with smoking cessation therapist; (also mixed nutrient supplementation) | Kinesiologist and dietitian | 4 weeks | Within one week of surgery | Usual care - education on the benefits of a healthy diet and physical activity but without specific information; received smoking cessation counseling if needed. |
| Fulop et al., 2021(36) | Multi | Smoking, PA, Diet, Alcohol | Home-based/ hospital (outpatients) | 30 mins supervised moderate intensity aerobic activity weekly & 30 minutes/day of jogging/walking & nutritional support & personalised advice for smoking and alcohol | Physiotherapist, nutritionist | 3-6 weeks | Day before surgery | Usual care |
| Furze et al, 2009 (66) | Single | PA | Home-based | 45-60 minute interview with nurse with follow-up telephone calls until operation. Relaxation techniques and goal setting for PA. | Nurse | NR | NR | Given verbal advice on risk factors |
| Gillis et al., 2014 (94), Chen et al., 2016 (52) | Multi | PA, Diet | Home-based | Up to 50 mins home-based aerobic and resistance exercise 3 days/week & personalized dietary counselling and whey protein supplementation. | Kinesiologist, dietitian, | 4 weeks | within 2 days before surgery | Same intervention post surgery |
| Goodman et al., 2008 (67) | Multi | PA, Diet | Home-based | Motivational interviewing and counselling for anxiety & lifestyle changes inc. advice regarding diet and exercise | Nurse | Average 9 months | Admission for surgery | Usual care |
| Goodney et al. 2017 (79) | Single | Smoking | Hospital (outpatient) | VBA, prescription for NRT and active referral to telephone-based smoking cessation counselling | Vascular surgeon | NR | NR | Usual care |
| Haddock et al. 1997 (68) | Single | Smoking | Hospital (outpatient) | Written information, NRT, diary to note down influences on smoking behaviour | Nurse | 1-2 weeks | Admission for surgery | Usual care |
| Hoogeboom et al 2010 (108) | Single | PA | Physiotherapy clinic & home-based | 60 mins supervised exercise (aerobic and resistance 2x/week for 3-6 weeks & encouraged to exercise at home and given pedometer | Physiotherapist | 3-6 weeks | within one week of surgery | Usual care |
| Karlsson et al, 2019 (109) | Single | PA | Home-based | 60 mins supervised home based exercise sessions (aerobic and resistance) 2-3x/week for at least 2 weeks & 150 mins unsupervised moderate physical activity | Physiotherapist | 2-3 weeks | NR | Usual care |
| Kim et al. 2009 (53) | Single | PA | Home-based | Aerobic exercise training; participants provided with cycle ergometer and training prescription; visited by physical therapist on several occasions | Self and physical therapist | 4 weeks | NR | Usual care; attention control |
| Kim et al. 2021 (80) | Single | PA | Medical Centre Therapy Pool | 60 min water therapy x3/week involving warm up, joint range of motion for flexibility and strength, low intensity endurance, cool down | Aquatic therapist | 4-8 weeks (median 29 days) | Immediately before surgery | Standard care and brochure on perioperative nutrition |
| Lai et al., 2017 (96) Interactive CardioVascular and Thoracic Surgery, China | Single | PA | Hospital (inpatient) | Aerobic exercise 30 mins/day | Physiotherapist | 1 week | Immediately before surgery | Usual care |
| Lai et al., 2017 (95) journal of surgical research, China | Single | PA | Hospital (inpatient) | aerobic exercise 30 mins/day for 7 days | Physiotherapist | 1 week | Immediately before surgery | Usual care |
| Lee et al., 2013 (54), Lee at al. 2015(55), Canada | Single | Smoking | Hospital (outpatient) | Brief counselling, brochures, referral to the Canadian Cancer Society’s Smokers’ Helpline, and 6 week NRT | Nurse | At least three weeks before surgery | Immediately before surgery | Usual care |
| Liang et al 2018 (81), Bernadi et al. (76), 2020 | Multi | PA, Diet | NR | Meetings included a four-part education on nutrition and making positive choices. Exercise programs included aerobic (e.g. walking, climbing stairs) and light anaerobic activity (e.g. group fitness) | Surgical specialists, Medical weight-loss experts, Dieticians, physical therapists, Health educators, Nurse practitioners, and Study coordinators | Up to 6 months | Immediately before surgery | Usual care |
| Licker et al., 2017 (97) | Single | PA | Hospital (outpatient) | HIIT & resistance training 2/3 times per week for 30 minutes | Physiotherapists | NR but median days to surgery was 26 | 2-4 days before surgery | Usual care |
| Ligibel et al. (82), 2016 | Single | PA | NR | 2 supervised 60-90 mins exercise sessions & counselling to achieve 220 mins of exercise/week at home and given a pedometer | Certified exercise trainers | NR but median days to surgery was 29 | Immediately before surgery | Mind–body control inc relaxation audio guide |
| Liljensoe et al. (85), 2019 | Single | Diet | NR | Low-energy liquid diet, nutritional education & weekly group sessions | Dietician | 8 weeks | 1 week before surgery | Usual care |
| Lindbäck et al., 2018 (98) | Single | PA | Physiotherapy clinic | General supervised exercise & behavioural therapy | Physiotherapists | 9 weeks | Immediately before surgery | Usual care |
| Lindstrom et al., 2008 (42) | Single | Smoking | NR | Weekly meetings/telephone counselling 1/week for 4 weeks, number to hot line providing smoking cessation advice, and NRT | Nurse | 4 weeks | None* | Usual care |
| Liu et al.(37), 2020, China | Multi | PA, Diet | Home-based & telephone | Aerobic and resistance exercise 30 mins 3 days/week, nutritional counselling | NR | 2 weeks | 1 day before surgery | Usual care |
| Lotzke et al.(86), 2019 | Single | PA | Spine clinic | Five 1-hour counselling sessions for physical activity behaviour change | Physical therapist | NR but intervention was 8-12 weeks pre-surgery | 1 week before surgery | Usual care |
| McHugh et al., 2001(69) | Multi | Smoking, PA, Diet, Alcohol | Home-based and GP practice | 1/month health education tailored sessions focusing on individual behaviours. | Nurse | NR but 8-8.5 months waiting list | NR | Usual care |
| McIsaac et al. 2022(56) | Single | PA | Home-based | 1 hour whole body strength, aerobic and flexibility exercises ≥ 3 x/week | NR | Mean = 39 days | Immediately before surgery | Received information on general PA and diet and a pedometer |
| Minnella et al., 2018(57) | Multi | PA, Diet | Home-based/ hospital (outpatients) | Individualised aerobic exercise & resistance exercise. One session face to face. Nutritional counselling. | Dietician, Kinesiologist | NR but median 36 days | Immediately before surgery | Usual care |
| Minnella et al., 2019(58) | Multi | PA, Diet | Hospital (outpatient) | Aerobic exercise & resistance training & dietary counselling. | Kinesiologist, Dietician, | NR but as least 4 weeks prior to surgery | NR | Usual care |
| Molenaar et al., 2023 (112) | Multi | PA, Diet, Smoking | Hospital (outpatient) | 1 hour aerobic and strength exercises 3x/week for 4 weeks; nutritional counselling; smoking cessation programme and NRT | Dietitian, Psychology trained personnel | 4 weeks | Immediately before surgery | Usual care |
| Moller et al., 2002(99), Villebro, 2008 (106) | Single | Smoking | NR | Individual counselling every week & NRT session. | Nurse | 6-8 weeks | NR | Usual care |
| Moug et al., 2019(70) | Single | PA | Home-based | 1 x consultation, behavioural counselling (& pedometer)to increase walking. 7 x phone calls. | Study coordinator | 13 weeks | 1-2 weeks before surgery | Usual care |
| Nguyen et al. 2022(87) | Multi | PA, Diet | Hospital- and home-based | 4 x 90 minute group-based, supervised sessions (2x/week) of education and multidisciplinary rehabilitation. Education materials on positive effects of exercise therapy before TKR, work rehabilitation, social support, diet and weight management, stress/anxiety management. Exercise therapy consisted of muscle strengthening, stretching, endurance training, proprioception exercises, walking and balance exercises, and home-based programme | Physiotherapist, instructor in physical activity, social worker, dietitian, psychologist, occupational therapist | 4 x 90 min sessions | NR | Usual care (provided with information booklet and standard advice from orthopaedic surgeon) |
| Nielsen et al., 2010(100) | Single | PA | Home-based | 30 min/day exercise aerobic and resistance training at home and two visits with physiotherapist to understand programme. | Physiotherapist | 6-8 weeks | Immediately before surgery | Usual care |
| Northgraves et al 2020(71) | Single | PA | University | 3x 60 min/week supervised aerobic and resistance exercise sessions. | Strength and conditioning instructor | NR but at least 2 weeks | Immediately before surgery | Advised to maintain normal exercise |
| Onerup et al., 2021(88) | Single | PA | Home-based & telephone | Advised to complete 30 mins aerobic activity ]]for 10-18 days. Encouraged to continue for 4 weeks post-op. Follow-up telephone call | Physiotherapist & Nurse | 10-18 days | NR | Usual care |
| Pehlivan et al. 2011 (84) | Single | PA | NR | Walking exercises on a treadmill 3x/day | NR | 1 week | Immediately before surgery | Usual care |
| Ratner et al. 2004 (59) | Single | Smoking | Hospital (outpatient) | 1x 15 min counselling, NRT, telephone hotline. | Nurse | 1-3 weeks | Within 24 hours of surgery | Usual care |
| Rengel et al (83). 2021 | Single | PA | Home-based & telephone | Weekly phone calls and personalised resistance training. | Physical therapist | 2-3 weeks | NR | Attention control with generalised health information |
| Rooks et al.(39), 2006 | Single | PA | Community fitness facility | Water and land-based aerobic and resistance exercise 3x/week for 6-weeks | Physiotherapist | 6 weeks | Immediately before surgery | Usual care |
| Rosenfeldt et al. 2011(101) | Single | PA | Hospital (outpatient) | 60 min supervised aerobic exercise 2x/week & >30 mins aerobic exercise on at least 2x/week. After initial 2 weeks 30 mins x 4 times/week of aerobic exercise at home. | Physiotherapist | NR (at least 2 weeks) | Immediately before surgery | Usual care |
| Sadr Azodi et al. 2008(43) | Single | Smoking | Hospital (outpatient)/ telephone | Meetings/telephone counselling 1x/week, number to hot line providing smoking cessation advice, and NRT | Nurse | 4 weeks | NR | Usual care |
| Santa-Mina et al. 2018(60) and Au et al. 2019(61) | Single | PA | Home-based | Individualised 'total body' exercise programme - aerobic and resistance, 60 min home based 3-4x/week. Information manual to support behaviour change strategies plus weekly phone or email contacts. | Study team | 4-8 weeks | Immediately before surgery | Attention control |
| Sawatzky et al. 2014(62) | Multi | PA, Diet | Medical fitness facility | 60 min exercise sessions ≥2 x/week & 12 class-based education sessions concerning medication use, exercise, stress, diet, and cardiovascular risk factor management | Cardiologists, nurses & exercise specialists | 2-16 weeks | NR only stated pre-surgery | Usual care |
| Sebio Garcia et al. 2017(102) | Single | PA | Hospital (outpatient) | 1 hour aerobic & resistance training 3-5x/week | NR | NR | None* | Usual care |
| Snowden et al. 2020 (72) | Single | Alcohol | Hospital (outpatient) | 5 mins structured advice relating to alcohol consumption, up to 25 mins brief behaviour change counselling, +/- booster session. | Health care professional | One-off session +/- booster session 6-8 weeks before surgery | 1-3 days pre-surgery | Usual care |
| Sorensen et al. 2003 (104) | Single | Smoking | Hospital (outpatient)/ telephone | Told to quit and given NRT and 2 x individual support sessions with nurse, option to call for additional support | Nurse | 2-3 weeks | Day before surgery | Maintain smoking habits |
| Sorensen et al. 2007 (103) | Single | Smoking | Hospital (outpatient)/ telephone | Patients allocated to 2 group telephone reminder group (1 month before surgery), outpatient 20min meeting & NRT | Nurse | At least 1 month before surgery | day of surgery | Usual care |
| Steffens et al. 2021 (110) | Single | PA | Hospital (outpatient) & home based | 1 hour supervised one-to-one aerobic and resistance training 1x/week & home-based functional exercises. Given fit bit and walk daily for 30 mins. | Physiotherapist | 2-6 weeks | Immediately before surgery | Usual care |
| Steinmetz et al. 2020 (105) | Single | PA | Ambulatory Prehabilitation | Supervised aerobic exercise 3x/week | NR | 2.5 - 3 weeks | One day before surgery | Usual care |
| Tew et al. 2017 (73) | Single | PA | Hospital (outpatient) | Supervised HIIT 3x/week | Research nurse, Physiotherapist, 2 Exercise scientists | 4 weeks | Immediately before surgery | Usual care |
| Thomsen et al. 2010 (44) | Single | Smoking | Hospital (outpatient) | 45-90 min counselling session with motivational interviewing & NRT. | Trained smoking cessation Counsellor | One-off session, 7 days before surgery | 2-days pre-surgery to 10 days post | Usual care |
| van der Velde et al. 2021 (111) | Multi | Smoking, PA, Diet, Alcohol | Mobile app | Be prepared app for education and self-monitoring. Received tailored information based on risk input. | Mobile App | 2 weeks | 3 days before surgery | Usual care |
| Waller et al. 2021 (74) | Multi | PA, Diet | Home-based | Given Fitbit Charge 2 and shown how to use app to increase PA. Weekly phone calls for tailored advice. | Physiotherapist; NR who delivered dietary advice | NR at least 2 weeks | Day before surgery | Given Fitbit Flex 2 - no screen providing feedback |
| Webb et al. 2022 (107) | Single | Smoking | Hospital-based | Registration with Quitline call-back service and posted NRT products | Quitline staff | NR but median time from listing to surgery was 99 days (IQR, 56– 194 days) for the intervention group | NR | Usual care (brochure on smoking and surgery) |
| Wolfenden et al. 2005 (89) | Single | Smoking | Hospital (outpatient)/ telephone | Computer based tailored counselling. Brief advice my HCP, one telephone counselling & NRT. | Computer, Nurse, Research assistant | One-off counselling session; 1-2 weeks NRT | >24 hour pre-surgery | Clinic staff could provide advice on quitting and prescribe pre- and postop NRT at their discretion |
| Wong et al 2017 (63) | Single | Smoking | Hospital (outpatient) | 1. structured 10-15 min counselling session, 2. pharmacotherapy, 3. educational pamphlet, 4. referral to telephone quitline | Anaesthesiologist or Pharmacist | 12 weeks | Immediately before surgery | Brief advice (3-5 mins) regarding smoking cessation and Quitline information for self-referral |

*they completed follow-up from before to post surgery.

**
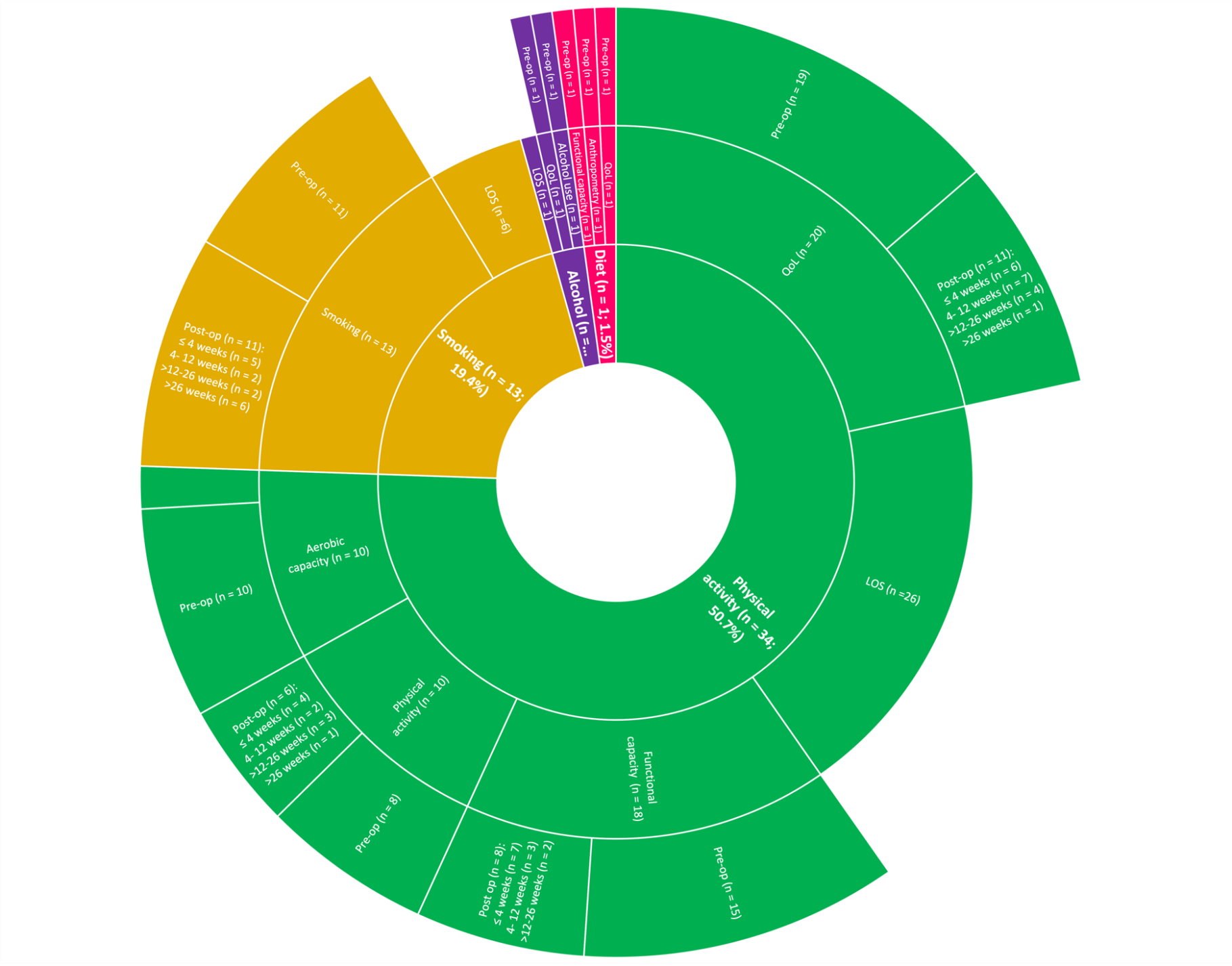
**

**Supplementary Figure 1.** Unimodal Prehabilitation interventions. The inner ring presents the health risk behaviour targeted in the intervention; the middle ring presents which outcomes were assessed, and outer ring presents the pre- and post-surgery timepoints at which these outcomes were assessed.


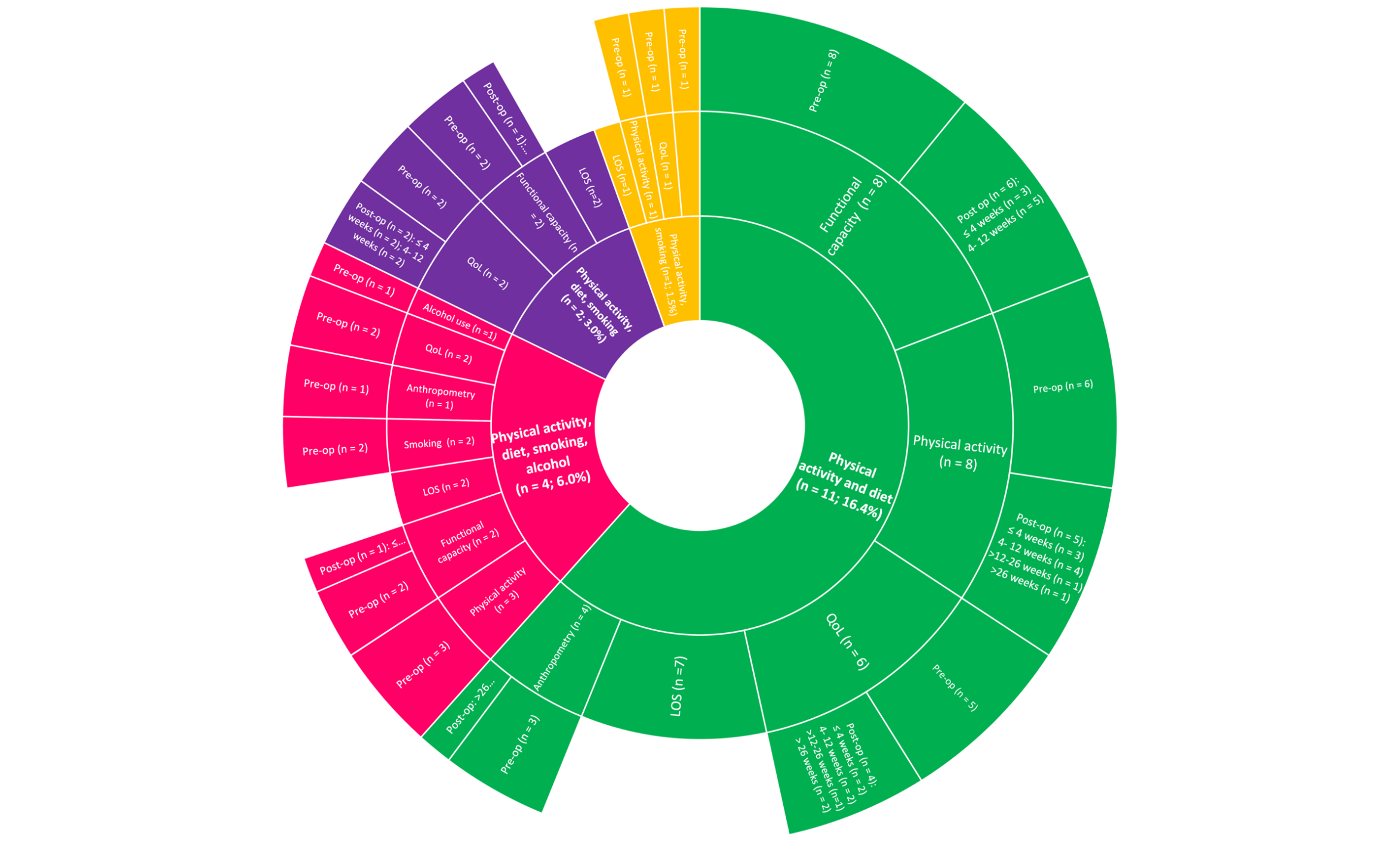


**Supplementary Figure 2:** Multimodal Prehabilitation interventions. The inner ring presents the health risk behaviour targeted in the intervention; the middle ring presents which outcomes were assessed, and outer ring presents the pre- and post-surgery timepoints at which these outcomes were assessed.


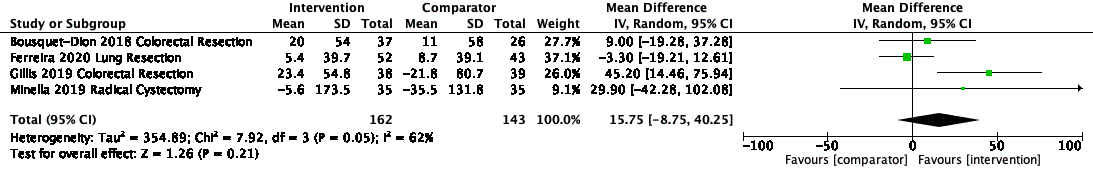


**Supplementary Figure 3:** Forest plot for 6MWT at 8 weeks post operatively


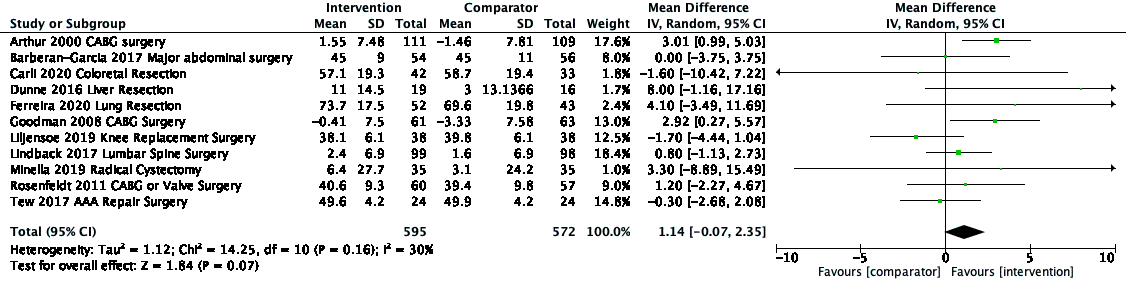


**Supplementary Figure 4:** Forest plot for Physical Component summary Scores (SF36) at pre surgery


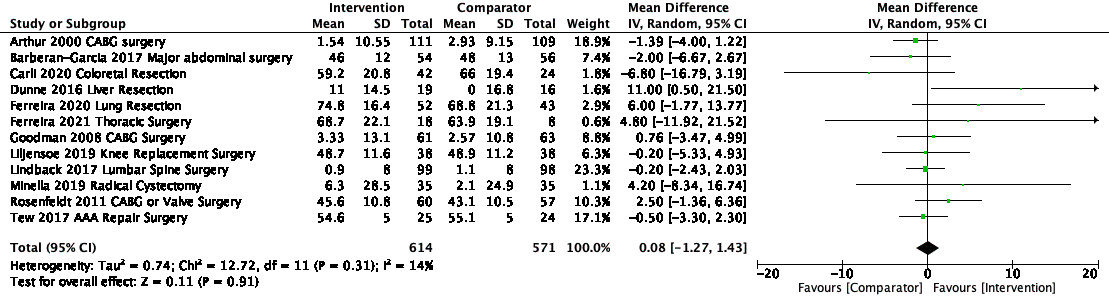


**Supplementary Figure 5:** Forest plot for Mental Component summary scores (SF36) at pre surgery


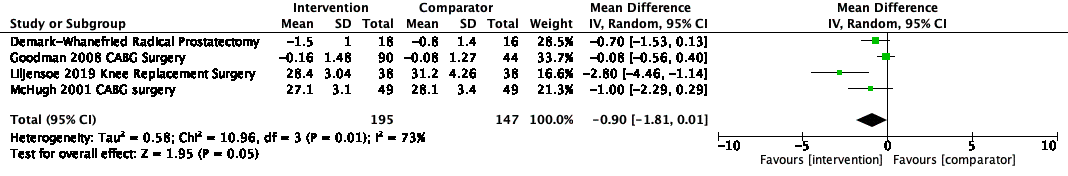


**Supplementary Figure 6:** Forest plot for BMI at pre-surgery


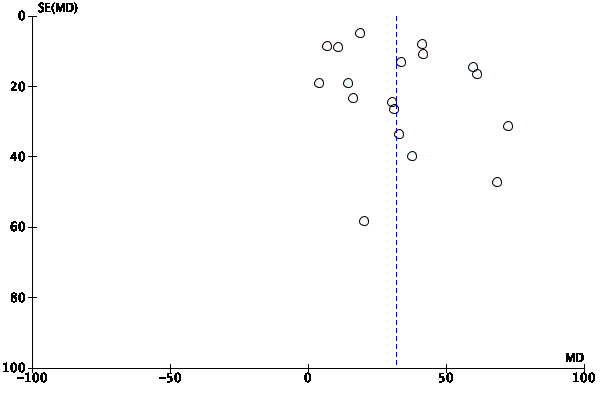


**Supplementary Figure 7.** Funnel plot of 6MWT pre surgery


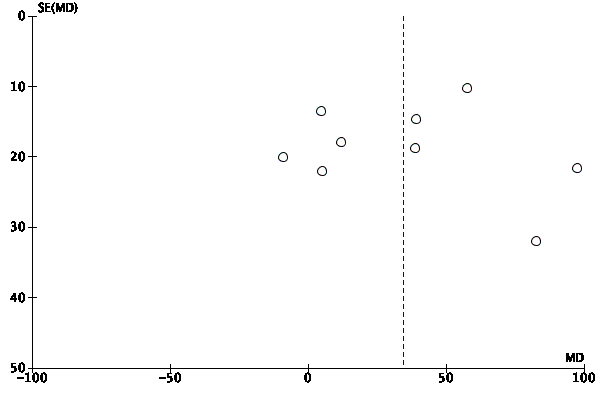


**Supplementary Figure 8.** Funnel plot 6MWT 4 weeks post-surgery


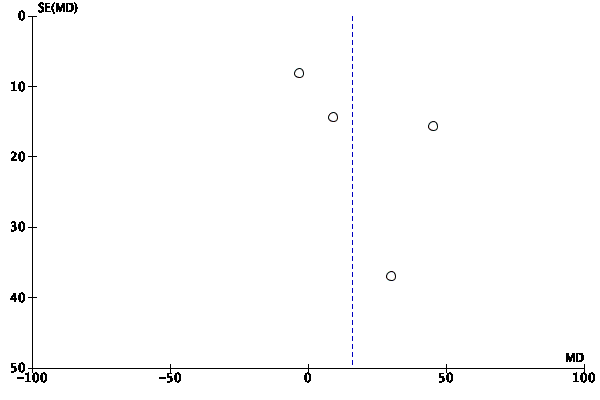


**Supplementary Figure 9.** Funnel plot 6MWT 8 weeks post-surgery


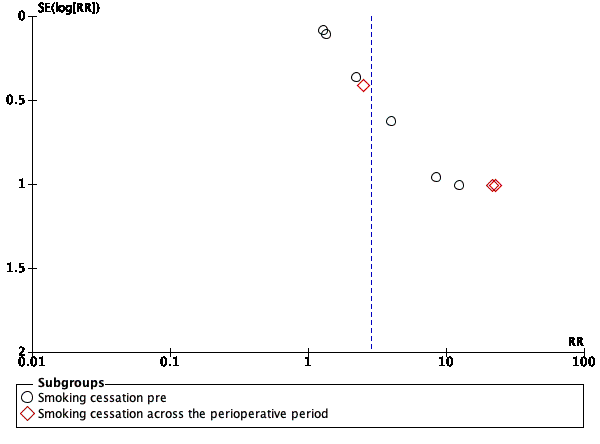


**Supplementary Figure 10.** Funnel plot of smoking pre-surgery

**
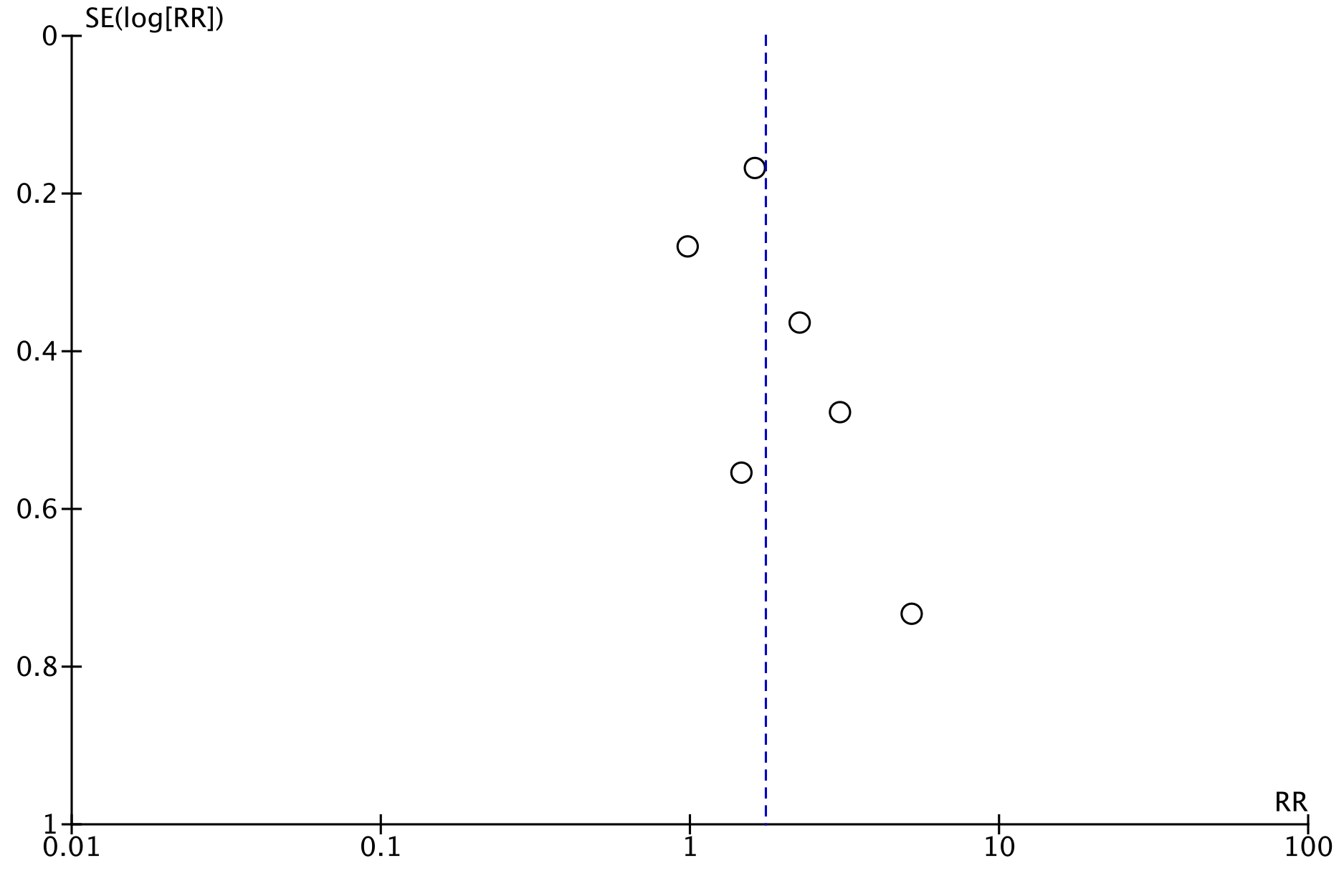
**

**Supplementary Figure 11.** Funnel plot of smoking 12 month post-surgery


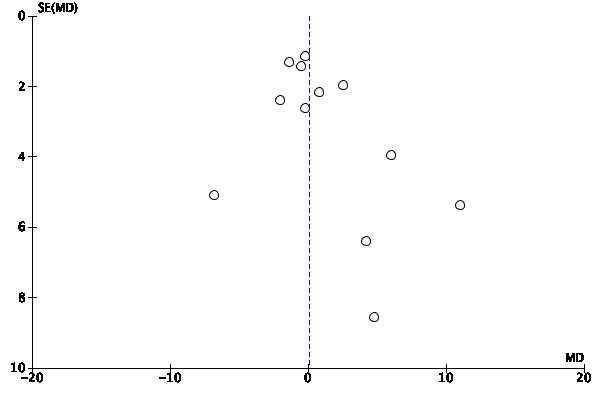


**Supplementary Figure 12.** Funnel plot of QoL MCS pre surgery


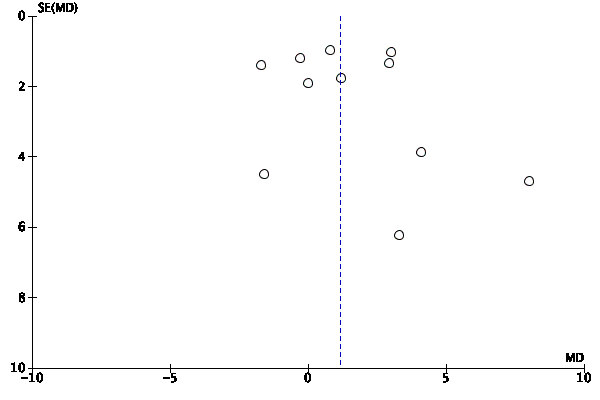


**Supplementary Figure 13.** Funnel plot QoL PCS pre surgery


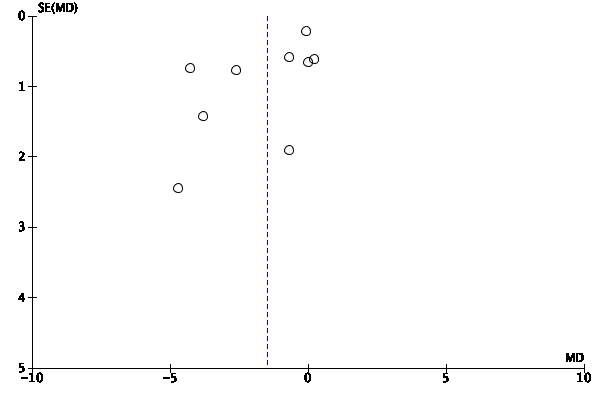


**Supplementary Figure 14.** Funnel plot LOS


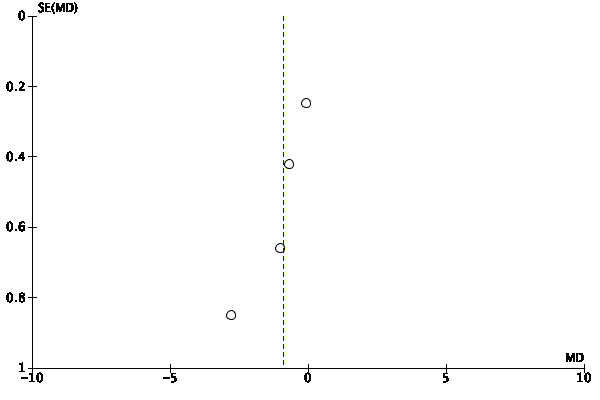


**Supplementary Figure 15.** Funnel Plot BMI
