# Supplementary material for: The effect of preoperative behaviour change interventions on pre- and post-surgery health behaviours, health outcomes, and health inequalities in adults: A systematic review and meta-analyses
Source: PLoS One. 2023 Jul 5;18(7):e0286757. doi: 10.1371/journal.pone.0286757 (PMC10321619; doi:10.1371/journal.pone.0286757)
Supplement: Attachment — Submitted filename: Response to reviewers.docx [file pone.0286757.s002.docx]

**Response to reviewer comments**

We are very grateful to both reviewers for taking the time and effort to review our manuscript. The comments were helpful and have made for a stronger study and paper.

Reviewer #1:

1. **Line 73: page 2 - four behavioural health risks - please describe these.**

These have now been identified individually*: “…that smoking, risky alcohol use, inadequate physical activity, poor diet (and resulting obesity) are leading causes of preventable ill-health globally…”*

2. **Line 78-79: Please explain the sentence "Behavioural modification.... with health inequalities - is there a ref for this?**

The link between behavioural modification and health inequalities is explained in the following sentences. To make this link clearer, we have joined them with the word ‘because’: *“Behavioural modification interventions in surgical populations also have the potential to reduce health inequalities because people from lower socioeconomic backgrounds are disproportionately represented in hospital and surgery populations, and…”*

3. **Line 89 - 92 - is a very general statement - this review would not be able to succinctly provide this information. My suggestion is to tone the message down.**

We have removed the sentence as we agree it is too general.

4. **The current aim of the paper is not clear - is it for health inequalities or health risk behaviours? Please clarify and discuss this appropriately.**

We agree and have changed it to*: “The primary aim of this review was to examine the effect of behavioural Prehabilitation interventions on pre- and post-surgery health outcomes and health behaviours across surgery specialities. The secondary aim was to examine these outcomes across the socioeconomic spectrum.”*

5. **Explain why the updated search was only done on Medline and not the other databases too.**

This review was very labour intensive and as most of the abstracts were found in Medline, it was decided to update the search only in Medline. We have added this to the manuscript.

6. **In the methods section - please indicate all the reviewers’ names for all searches, screens and conflict resolution**.

We have entered the initials in all the above.

7. **Line 150; Explain LOS, 6 MWT, QOL, and BMI - these are abbreviations.**
   We have previously defined these abbreviations in full in earlier sections of the paper hence why only abbreviations are used. However, if the editor would rather us write in full throughout, we can do so.
8. **Under study characteristic – list refs. (N=19), N=28, Same for the intervention characteristics – list the ref of the articles discussed**

We have completed this.

9. **Line 402 – explain why Weight loss was discussed when it was not one of the outcomes defined in the health behaviour list. Ref 32 – 2 full stops in the ref format.**

Thank you and we have realised that this has been missed even though it was in the PICO table, we have ensured that weight loss is mentioned and included throughout, including a rationale in the introduction *“…(i.e. arises from poor diet and inadequate physical activity therefore relevant to the scope of this work).”*

10. **Supplementary table 1 – for the weight loss portion the Amendment and the rationale do not match – the amendment suggested looking at intervention promoting weight gain but the rationale suggests it was done otherwise. Please clarify.**

We agree that the phrasing used to describe this amendment was not clear and consistent with the rationale. The amendment has been changed to*: “We excluded extraction of anthropometric or dietary outcomes where the intervention aimed to promote weight maintenance/gain (not weight loss).”*

11. **Sup table 2 – NACRT not defined in legends**

The definition has now been included in the Supp Table 2 legend.

Reviewer #2: This is a very interesting systematic review (SR) and metaanalysis (MA) about health behavioural interventions delivered during prehabilitation across different surgical specialties. The SR is well conducted and report some interesting findings and the manuscript is very well written and easy to read. I do have, however, some comments that I would like the authors to clarify:

1**. The search was conducted more than 6 months ago. With more than 20 papers being published about prehabilitation each month, I believe the search should have been updated. At least, monthly alerts should have been set to be up to date in the latest publications. As this is not reported in the methods, I wonder whether this was the case for this review**.

That is a great idea about monthly alerts, and we will take this forward in our work. We updated the search again on 30^th^ March 2023 and found one additional study (Molenaar et al., 2023) which we have included in the review and the results have been incorporated into the results. As the outcomes were reported as medians (IQR), we contacted the authors to request the means (SD) to be able to include the data in the meta-analysis. However, we did not hear back from them and were unable to do so.

**2. The range for duration of intervention was from one week to 9 months. Was this taken into consideration when performing the meta-analyses? Duration of intervention is important in behavioural interventions such as dietary intake and physical activity, thus this should be taken into account when performing the MA beyond the random model. Have the authors conducted any subanalysis by duration of prehab? This might be interest in some outcomes like LOS, 6MWT or BMI and may reduce heterogeneity when present.**

One of the issues we found and something that needs taking forwards in prehabilitation studies is that the duration of the intervention was not often reported, and it was very difficult to assess the duration of the intervention e.g., some said at least 2 weeks before surgery but did not report actual length of intervention. For the 6MWT there were two interventions over 8 weeks and one intervention that lasted between 2-16 weeks. The other interventions were either not reported or lasted between 1-4 weeks. We have added the following*: “The duration of the interventions ranged from one week to 16 weeks, with three trials not reporting duration and some reporting a range, thus limiting our ability to examine the duration of intervention on outcomes.”*

**3. Although the type of surgery is indicated in the description of the trials, I also think this information should be included somehow in the metaanalysis. I've noticed that most studies included in the MA for LOS were performed in patients undergoing lung cancer surgery. As for duration of intervention, type of surgery night influence the results of prehabilitation on LOS, as some in some surgeries the impact of nutrition and functional capacity might be greater than in others to reduce LOS.**

To help the reader we have added the type of surgery in the legends on the meta-analysis. We also examined the LOS MA specifically and half the studies were for lung cancer surgery. We have added the following and updated the discussion: *“In a post-hoc sensitivity analysis focusing on the type of surgery and LOS, only lung cancer surgery (n=4) was associated with a significant difference of -3.6 days (95% CI -4.5 to -2.6, p<0.001, I^2^ 0%). As there were less than two of each other surgical specialty we combined them and there was no difference in LOS (-0.1 days, 95% CI -0.5 to 0.3, p=0.59, I^2^ 0%).”*

For the other MA we explored whether we could conduct any sensitivity analyses but there was not the majority of surgical specialties that we could divide into. The only option was 6MWT but in a sensitivity analysis there were no differences by surgical specialty.

**4. I would like the authors to clarify what they consider Physical Activity in this paper, because I feel the SR includes a mix of exercise-based interventions (some supervise) and physical activity promotion which are two different things that lead to different results. Could the authors please elaborate in the methods section what do you consider by a physical activity intervention during prehabilitation?**

Physical activity is an umbrella concept that includes several subtypes including exercise i.e., planned, structured, and repetitive and has as a final or an intermediate objective the improvement or maintenance of physical fitness (Caspersen et al. Physical activity, exercise, and physical fitness: definitions and distinctions for health-related research. Public Health Rep. 1985). In theory, all subtypes from incidental activity or structured exercise on a bike have the same aim of increasing cardio-respiratory fitness to improve the resilience for surgery, and both can also promote regular, physical activity over the longer-term. We have included a sentence in the Methods to make clear that we were interested in interventions that targeted any subtype of physical activity for these reasons: *“We included physical activity interventions that targeted any subtype of physical activity e.g. (both supervised and non-supervised) exercise (Caspersen et al., 1985) as in theory,* *all subtypes have the same aim of increasing cardio-respiratory fitness to improve the resilience for surgery, and can also promote regular, physical activity over the longer-term.”*

**5. There is one subanalysis from the main study by Barberan-Garcia et al. that was not included in the review and should have been, as it includes information regarding post-surgery functional capacity and physical activity levels. The reference for the study is Barberan-Garcia et al. BJA 2019, 123(4):450-456.**

Thank you, we have included this study in our review and the results have been incorporated into the narrative synthesis. Unfortunately, we cannot add the results to the meta-analysis for functional capacity as we synthesised 6MWT which this study did not report. Also, there was not enough studies to meta-analyse quality of life measures post-surgery.

**6. Why only studies that reported LOS in mean and not median were included in the MA? As you know, mean and SD can be calculated from median and 25-75 quartiles.**

We understand the means can be calculated using medians, however, if medians are reported it is likely data are not normally distributed. The underlying effects according to the Cochrane handbook for the random effects meta-analysis should be normally distributed therefore we are reluctant to calculate means from medians.
